# Severe inflammation in new-borns induces long-term cognitive impairment by activation of IL-1β/KCC2 signaling during early development

**DOI:** 10.1186/s12916-022-02434-w

**Published:** 2022-07-27

**Authors:** Donghang Zhang, Yujiao Yang, Yaoxin Yang, Jin Liu, Tao Zhu, Han Huang, Cheng Zhou

**Affiliations:** 1 Department of Anesthesiology, West China Hospital of Sichuan University, Chengdu, 610041 China; 2 Laboratory of Anesthesia and Critical Care Medicine, National-Local Joint Engineering Research Centre of Translational Medicine of Anesthesiology, West China Hospital of Sichuan University, Chengdu, 610041 China; 3 Department of Anesthesiology, Affiliated Hospital of North Sichuan Medical College, Nanchong, 637000 China; 4 Department of Anesthesiology & Key Laboratory of Birth Defects and Related Diseases of Women and Children, Ministry of Education, West China Second Hospital of Sichuan University, Chengdu, 610041 China

**Keywords:** Cognitive impairment, GABAergic shift, IL-1β, KCC2, Neonatal inflammation, Sepsis

## Abstract

**Background:**

Neonatal sepsis can induce long-term cognitive impairment in adolescence or adulthood, but the underlying molecular mechanism is not fully understood. The expression of K^+^-Cl^–^ co-transporter 2 (KCC2) plays a pivotal role in the GABAergic shift from depolarizing to hyperpolarizing during early postnatal development. In this study, we aimed to determine whether neonatal severe inflammation-induced cognitive impairment was associated with the expression of KCC2 during early development.

**Methods:**

Neonatal severe inflammation was established by intraperitoneal injection of high dose lipopolysaccharide (LPS, 1 mg kg^–1^) in postnatal day 3 (P3) rats. The Morris water maze task and fear conditioning test were used to investigate long-term cognitive functions. ELISA, RT-PCR and Western blotting were used to examine the expression levels of proinflammatory cytokines and KCC2. Perforated patch-clamping recordings were used to determine the GABAergic shift.

**Results:**

Neonatal severe inflammation led to long-term cognitive impairment in rats. Meanwhile, sustained elevation of interleukin-1 beta (IL-1β) levels was found in the hippocampus until P30 after LPS injection. Elevated expression of KCC2 and hyperpolarized GABA reversal potential (E_GABA_) were observed in CA1 hippocampal pyramidal neurons from the P7-P10 and P14-P16 rats after LPS injection. Specific knockdown of IL-1β mRNA expression rescued the elevated expression of KCC2 and the hyperpolarized E_GABA_ at P7-P10 and P14-P16. Accordingly, specific knockdown of IL-1β or KCC2 expression improved the cognitive impairment induced by neonatal severe inflammation.

**Conclusions:**

Sustained elevation of IL-1β in the hippocampus may induce cognitive impairment by upregulation of KCC2 during early development.

**Supplementary Information:**

The online version contains supplementary material available at 10.1186/s12916-022-02434-w.

## Background

Sepsis is a life-threatening syndrome resulting from a dysregulated host response to infections [1], particularly in new-borns. Neonatal sepsis is usually caused by bacterial invasion into the bloodstream within the first month of life [2], which is a major cause of mortality in neonatal intensive care units [3, 4]. The World Health Organization estimates that neonatal sepsis causes one million deaths per year worldwide, and 42% of those deaths occur in the first week after birth [5]. In recent years, the survival rate of neonatal sepsis has markedly improved with medical advances. Unfortunately, neonatal sepsis survivors have an increased risk of long-term cognitive impairments [6–8]. However, the molecular mechanism by which neonatal sepsis induces long-term cognitive impairment remains unclear.

During sepsis, the expression levels of proinflammatory cytokines, such as TNF, IL-6 and IL-1β, are increased in the central nervous system (CNS), which is believed to play a pivotal role in long-term cognitive impairment after sepsis [9, 10]. Systemic injection of lipopolysaccharide (LPS), a bacterial endotoxin, is commonly used to induce inflammation in neonatal animals to reproduce the multiple complications, such as cognitive impairment, which are also observed in human new-borns after sepsis [11–14]. Injection of LPS can induce increased proinflammatory cytokines, including TNF, IL-6 and IL-1β [15]. In particular, IL-1β plays a pivotal role in sustained neuroinflammation after sepsis and is closely implicated in memory processing and long-term potentiation, as well as neonatal sepsis-induced cognitive impairment [10, 16, 17]. However, how IL-1β mediates neonatal sepsis-induced cognitive impairment, particularly during the developing period of the CNS, remains unclear.

γ-Aminobutyric acid (GABA) is the major inhibitory neurotransmitter in the CNS. Interestingly, GABA mediates the depolarizing effects in the early developmental stage of various parts of the vertebrate CNS due to a high intracellular concentration of chloride maintained by an importer, Na^+^-K^+^-2Cl^–^ co-transporter 1 (NKCC1) [18]. The depolarizing actions of GABA play an important role involving cell proliferation and survival, migration, differentiation, and early network wiring [18]. In recent years, emerging evidence put insight into the role of depolarizing GABA signaling in vivo [18, 19], which may be region-dependent. For example, depolarizing effects of GABAergic transmission mediate excitatory modulation in mouse hippocampus [19], whereas it causes inhibitory effects [18–20] in mouse neocortex during early postnatal development. During postnatal development, a shift from depolarizing to hyperpolarizing effects of GABAergic activation was induced by enhanced chloride extrusion mediating by upregulation of K^+^-Cl^–^ co-transporter 2 (KCC2) [21–24]. This developmental GABAergic shift can serve as an indicator of the stage of maturation of distinct neuronal populations [25], and is associated with synaptic development and neuronal plasticity [26]. Besides setting the polarity of GABAergic function during neuronal maturation, KCC2 has profound ion transport-independent functions, such as modulating developmental apoptosis and early network activities, and is implicated in several diseases [18, 27–29].

Although previous studies showed that IL-1β can regulate the expression of KCC2 in the CNS [27, 30], it is unknown whether abnormal GABAergic shift induced by altered expression of KCC2 is involved in neonatal sepsis- or severe inflammation-induced long-term cognitive impairment. Herein, we hypothesized that sustained elevation of IL-1β levels affected the GABAergic shift by modulating KCC2 expression in CA1 hippocampal pyramidal neurons during the development period, which finally contributed to long-term cognitive impairment after neonatal severe inflammation.

## Methods

### Animals

The experimental protocol was approved by the Animal Ethics Committee of West China Hospital of Sichuan University (Chengdu, Sichuan, China) and was conducted in accordance with the Animal Research: Reporting of In Vivo Experiments (ARRIVE) guidelines. Sprague–Dawley rats on gestational day 16 were purchased (Chengdu Dossy Experimental Animals CO., LTD.) and were separated and monitored for the offspring’s birth day. Postnatal offspring (both sexes) were kept with their mothers with food and water available ad libitum. Animals were maintained under a 12-h (7:00 to 19:00) light/dark cycle at a constant humidity (45%-55%) and temperature (22–24 °C). After weaning at P21, animals were housed in groups of five rats per cage.

### Injection materials

LPS (Sigma, USA) was dissolved in normal saline and injected intraperitoneally with dose of 1 mg kg^−1^ at P3. IL-1β-siRNA (sense sequence: 5’-GCACAGACCUGUCUUCCUATT-3’; antisense sequence: 5’-UAGGAAGACAGGUCUGUGCTT-3’), with the modification of 5’-FAM, KCC2-siRNA (sense sequence: 5’-GCCAUUUCCAUGAGCGCAATT-3’; antisense sequence: 5’-UUGCGCUCAUGGAAAUGGCTT-3’) with the modification of 5’-CY5, and negative control (Control-siRNA, sense sequence: 5’-UUCUCCGAACGUGUCACGUTT-3’; antisense sequence: 5’-ACGUGACACGUUCGGAGAATT-3’) (GenePharma, Shanghai, China) were dissolved in RNase-free water. IL-1β-siRNA, KCC2-siRNA or negative control was mixed with In vivo SilenceMag™ transfection reagent (OZ Biosciences, Marseille, France) to a final concentration of 1 μg μL^−1^ 20 min before injection. Then, IL-1β-siRNA, KCC2-siRNA or negative control was injected into the bilateral CA1 regions of the hippocampus (0.5 μL for each side) at P2 and/or P7.

### Stereotaxic injection

Rats were placed on ice to induce hypothermia anesthesia as described in a previous study [31] and mounted in a stereotaxic apparatus (RWD, Shenzhen, China). Ophthalmic ointment was applied to the eyes of P7 rats. IL-1β-siRNA/KCC2-siRNA/control-siRNA (0.5 μL) was bilaterally injected into the hippocampal CA1 regions (midpoint between the bregma and sagittal suture, lateral: ± 1.5 mm, depth: 1.2–1.4 mm for P2 and 1.6–1.8 mm for P7) at a rate of 100 nL min^−1^. After completion of the injection, the glass pipette was left in place for 5 min and withdrawn slowly to avoid backflow. Then, the animals were allowed to recover on a heating blanket before returning to their home cages.

### Neonatal severe inflammation model and grouping

To minimize litter effects, postnatal offspring of both sexes from each litter were randomly assigned into the experimental groups. Pups were then returned to their dams and weaned until P21. After P21, the rats were gathered into each experimental group and randomly housed 5 rats per cage (the same sex). Therefore, the rats in each cage were from differentially random litters. All animal assignments were done to ensure the approximately equal distribution of sex and treatment from each litter. No different sets of litters were used for the various cohorts of experiments. The neonatal severe inflammation model was induced by intraperitoneal injection of a high dose of LPS (1 mg kg^−1^) in P3 rats. In the control group, rats received an intraperitoneal injection of normal saline (NS) alone. In the LPS group, rats received an intraperitoneal injection of LPS alone. In the NS + control-siRNA group, rats received hippocampal CA1 injection of control-siRNA and intraperitoneal injection of normal saline. In the LPS + control-siRNA group, rats received hippocampal CA1 injection of control-siRNA and intraperitoneal injection of LPS. In the LPS + IL-1β-siRNA group, rats received hippocampal CA1 injection of IL-1β-siRNA and intraperitoneal injection of LPS. In the LPS + KCC2-siRNA group, rats received hippocampal CA1 injection of KCC2-siRNA and intraperitoneal injection of LPS.

### Morris water maze test

Spatial learning and memory of adolescent rats were assessed by the Morris water maze test as described previously [32]. Briefly, the system consisted of a round pool (90 cm diameter, 50 cm depth) divided into four quadrants. Different-shaped objects were attached to the wall of each quadrant to serve as spatial visual clues. The temperature of the water was maintained at 30 ± 1 °C. A circular platform with a diameter of 10 cm was placed 1 cm below the surface of black water and 30 cm away from the pool wall. The quadrant containing the platform was defined as the target quadrant. The swimming traces of the rats were recorded by an automatic video camera. Before the orientation navigation test, every rat was trained three times a day for 4 consecutive days. The rat was placed into the water with face to the pool wall. If the rat found the platform within 90 s and stayed on the platform for 15 s, the time period was defined as the escape latency. Otherwise, the rat was guided to the platform to stay for 15 s, and the escape latency was recorded as 90 s. During the test, the platform was removed, and the rat was allowed to swim for 90 s. The number of crossings in the target area, the time spent and the total distance travelled in the target quadrant, as well as the mean speed, were recorded. SMART software (Panlab, Barcelona, Spain) was used to analyse the swimming trace of each rat.

### Fear-conditioning test

Two days after Morris water maze, the same rats were subjected to the fear conditioning test according to the paradigm as described previously with minor modifications [33]. Rats were trained to connect the context (chamber) with an aversive stimulus (foot shock; unconditioned stimulus, US), which can be used to assess hippocampal-dependent contextual fear conditioning. The foot shock was also paired with a tone cue (conditioned stimulus, CS) to assess hippocampal-independent cued fear conditioning. Conditioned fear was displayed as a freezing behaviour by ceasing all movement except for respiration when rats were re-exposed to the context or the tone. Training parameters were as follows: tone, 30 s, 80 dB, 2 kHz; shock, 2 s, 0.8 mA. On day 1, each rat was placed into a fear conditioning chamber and allowed to explore freely for 2 min. Then, a tone was delivered followed by a foot shock. Two minutes later, a second CS-US pair was delivered. On day 2, each rat was re-exposed to the same fear conditioning chamber but without delivery of a CS or foot shock. Freezing was recorded for 3 min. One hour later, each rat was placed in a new context containing a different odor, cleaning solution, floor texture, and chamber wall. The rats were allowed to explore for 2 min before being re-exposed to the tone. Freezing was assessed for 3 min and then measured using the ANY-maze video tracking system and software (Stoelting, Wood Dale, IL).

### Western blot

Rats were anesthetized with pentobarbital sodium (100 mg kg^−1^) and transcardially perfused with ice-cold Ringer's solution. The hippocampus was quickly removed and homogenized with ice-cold lysis buffer (Beyotime, China) containing phosphatase and protease inhibitors. The protein concentration was determined by a BCA Protein Assay Kit (Beyotime, China). Twenty micrograms of protein samples were separated by a NuPAGETM4-12% Bis–Tris Gel (Thermo Fisher Scientific) and transferred to polyvinylidene difluoride membranes (Thermo Fisher Scientific) using the iBLOT2 system. The membrane was blocked in Tris-buffered saline containing 0.1% Tween-20 and 5% non-fat milk for 2 h and then incubated with primary antibodies at 4 °C overnight, including rabbit anti-IL-1β (1:2000, Abcam), rabbit anti-KCC2 (1:1000, Sigma-Aldrich), and rabbit anti-β-actin (1:1000, Proteintech). Then, the membranes were incubated with horseradish peroxidase-conjugated anti-rabbit (1:5000, Proteintech, China) for 1 h at room temperature and scanned with chemiluminescence reagents (ECL; Amersham Pharmacia Biotech, Piscataway, NJ) using the Chemidoc XRS system (Bio-Rad). The density of the bands was analysed by ImageJ software. The density of the bands from the control group was set as 100%. The relative density values from the other groups were determined by dividing the density values from these groups by the control values after each was normalized to the β-actin. Original images for Western blotting results were shown in Additional file 1.

### Real-time PCR

Total RNA of the hippocampus was isolated using a Eastep® Super RNA extraction kit (Promega, Shanghai, China) followed by reverse transcription with a GoScript™ Reverse Transcription Kit (Promega, Shanghai, China) according to the manufacturer’s protocol. Finally, RT-PCR was performed with GoTaq® qPCR Master Mix (Promega, Shanghai, China) and specific primers (Sangon Biotech, Shanghai, China). The relative fold change in gene expression was calculated with the 2^−ΔΔCt^ method with GAPDH as the internal control [34]. The primers used to detect TNF, IL-6, IL-1β, KCC2, and GAPDH mRNA were as follows:

- TNF forward: 5’-CTGTGAAGGGAATGGGTGTT-3’;
- TNF reverse: 5’-CAGGGAAGAATCTGGAAAGGTC-3’;
- IL-6 forward: 5’-GGCCCTTGCTTTCTCTTCG-3’;
- IL-6 reverse: 5’-ATAATAAAGTTTTGATTATGT-3’;
- IL-1β forward: 5’-AGTTGACGGACCCAAAAG-3’;
- IL-1β reverse: 5’-AGCTGGATGCTCTCATCAGG-3’;
- KCC2 forward: 5’- AGGTGGAAGTCGTGGAGATG-3’;
- KCC2 reverse: 5’-CGAGTGTTGGCTGGATTCTT-3’;
- GAPDH forward: 5’-GACATGCCGCCTGGAGAAAC-3’;
- GAPDH reverse: 5’-AGCCCAGGATGCCCTTTAGT-3’.

### Enzyme-linked immunosorbent assay (ELISA)

ELISA experiments were performed to quantify the levels of proinflammatory cytokines, including TNF, IL-1β, and IL-6, in the blood serum. Briefly, rats were anesthetized with ~ 3% sevoflurane. Blood samples (200–500 μL) were collected directly from the heart, preserved in EDTA tubes, and incubated for at least 30 min at room temperature. Then, the samples were centrifuged at 2000 g for 20 min at room temperature to separate the serum from the cellular blood components. The supernatants were immediately extracted and frozen in liquid nitrogen. ELISA kits (Neobioscience) were used to determine cytokine levels according to the manufacturer’s instructions. Absorbance at 450 nm was measured with a Tecan Sunrise™- microplate reader with a wavelength correction at 680 nm connected to Magellan software. The protein concentration of the samples was determined by the measured optical density of the reaction according to the optical density of the known standard samples.

### Brain slice preparation

Rats at P7-P10, P14-P16, or P28-P32 of both sexes were anesthetized with pentobarbital sodium (100 mg kg^−1^). The brain was quickly dissected, and transverse dorsal hippocampal slices (300 μm in thickness) were obtained in ice-cold sucrose-based artificial cerebrospinal fluid containing (in mM): 260 sucrose, 26 NaHCO_3_, 3 KCl, 1.25 NaH_2_PO_4_, 1 CaCl_2_, 5 MgCl_2_, and 10 glucose using a vibratome (VT1000 A; Leica). The slices were immediately transferred and incubated at 35 °C with artificial cerebrospinal fluid containing (in mM): 130 NaCl, 3 KCl, 2 MgCl_2_, 2 CaCl_2_, 1.25 NaH_2_PO_4_, 26 NaHCO_3_, and 10 glucose for 45 min and then maintained at room temperature (24–26 °C) for 30 min before recording. The slicing and incubation solution were continuously bubbled with 95% O_2_/5% CO_2_, with pH at 7.35.

### Perforated patch-clamping recording

Hippocampal slices were mounted in a recording chamber and perfused with artificial cerebrospinal fluid (aCSF) at a flow rate of 2 ~ 3 ml min^−1^ and bubbled with 95% O_2_ and 5% CO_2_, pH = 7.35. CA1 pyramidal cells were probed sequentially starting near the CA2/CA1 border and proceeding medially at well-separated locations. Pyramidal neurons were then identified under differential contrast/infrared illumination by their location in the cell body layer and by their pyramidal shape. Perforated recordings were made using patch pipettes (6–8 MΩ) filled with the internal solution containing (in mM) 140 K-gluconate, 10 HEPES, 5 EGTA 1 MgCl_2_, 2 Na_2_-ATP, 0.3 Na_2_-GTP, pH adjusted to 7.2 with KOH and osmolarity to ~ 285 mOsm. Patch pipettes were minimally front-filled with the standard internal solution and were then backfilled with gramicidin-containing solution. Gramicidin (HY-P0163, MedChemExpress), dissolved in dimethyl sulfoxide (DMSO) at a final concentration of 50 μg mL^−1^, was used as the pore-forming agent for perforated recordings. Gramicidin channels are selectively permeable to monovalent cations and small neutral molecules but impermeable to chloride, which enables electrical access to recorded neurons without disrupting their anionic gradients. Within ~ 20–40 min after giga seal formation, the access resistance slowly dropped and stabilized at ~ 20–35 MΩ. Resting membrane potential (RMP) was then recorded as the voltage with no injected current. To estimate the chloride concentration, the GABA reversal potential (E_GABA_) was evaluated. Neurons were held at –60 mV, and the membrane potential was stepped to various test potentials from –80 to –30 mV. During each membrane potential step, GABA (10 μM) in extracellular solution was delivered by bath-application to activate currents in the presence of cyanquixaline (CNQX, 10 μM) and DL-2-amino-5-phosphonopentanoic acid (40 μM). A linear regression between amplitude of GABA-induced currents versus membrane potential was calculated, and the intercept of this line with the abscissa was taken as E_GABA_. All the electrophysiological recordings were conducted using an Axopatch 700B amplifier and Digidata1440 digitizer linked to a computer running pClamp 10.2 software (Molecular Devices, Sunnyvale, USA). Signals were sampled at 20 kHz and filtered at 10 kHz. Cell and electrode capacitances were compensated electronically during recording. The cell was discarded if the access resistance changed by > 25%.

### Statistical analysis

Data were expressed as the means ± standard deviation (SD), and statistical analyses were performed using GraphPad Prism 8.0 software (GraphPad Software, CA, USA). Normality of data distribution was assessed using the Shapiro–Wilk test. Paired/unpaired Student’s t tests or Mann–Whitney U tests were used for comparisons of parametric distribution data or nonparametric distribution data between two groups, respectively. Data from three or more groups were analysed using a one-way or two-way analysis of variance (ANOVA) with repeated measures followed by a Bonferroni or Tukey post hoc test. The exact analysis used for each comparison was described in the figure legends, and all the statistical information for each result were summarized in Additional file 2: Table S1-S11. *P* < 0.05 was considered statistically significant.

## Results

### Neonatal severe inflammation leads to long-term cognitive impairment in adolescent rats

**Fig. 1:**
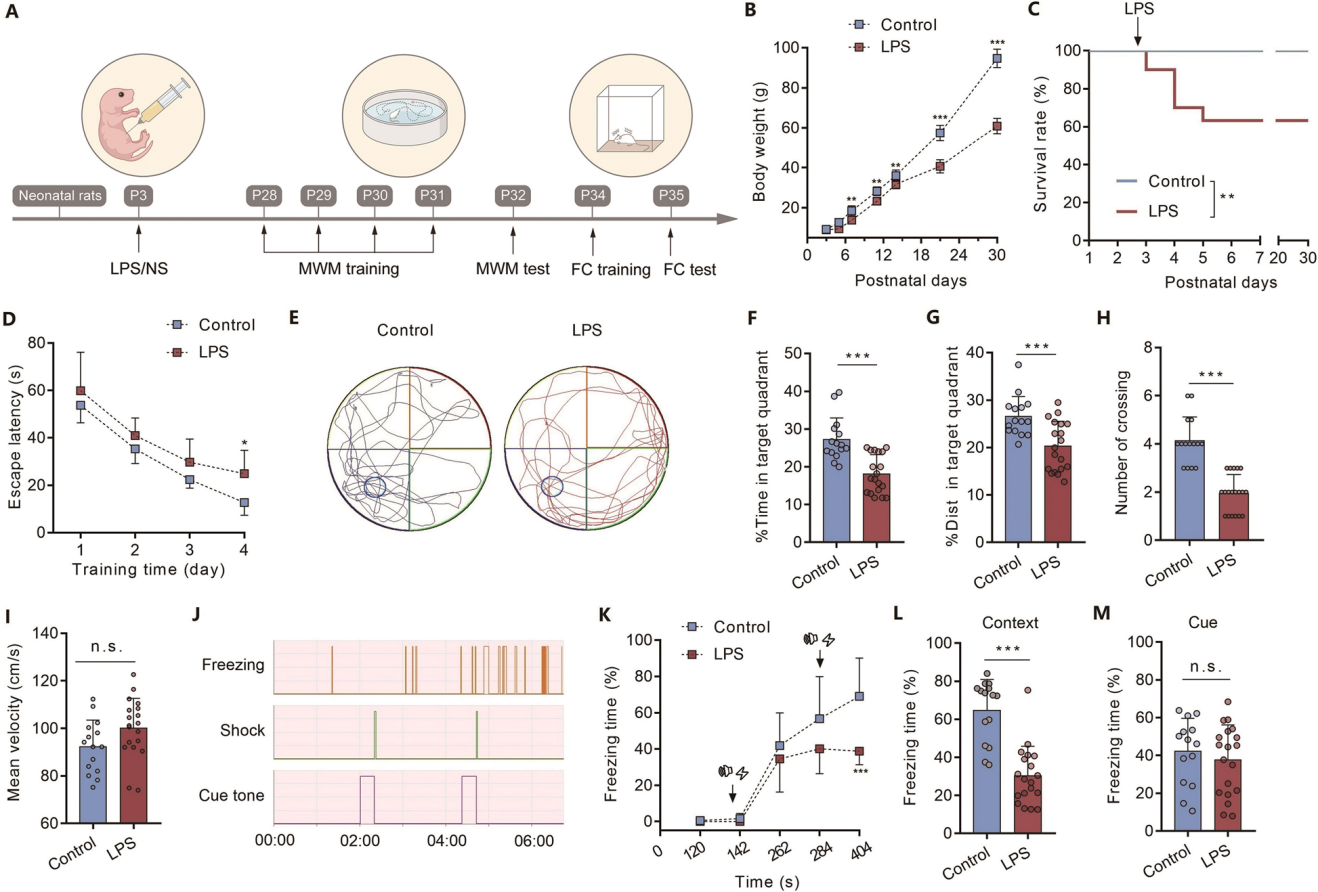
Neonatal severe inflammation leads to long-lasting cognitive impairment in adolescent rats. **(A)** Schematic illustrating the chronological order used for the establishment of the inflammation model and cognitive testing. Five litters were used in this cohort of experiment. **(B)** The development of body weight in rats (*n* = 8). **(C)** The survival curve of rats. **(D)** Learning curve for the escape latency. **(E)** Representative traces for the MWM test. **(F)** The time spent in the target quadrant (*n* = 15–19). **(G)** Distance spent in the target quadrant (*n* = 15–19). **(H)** Number of platform crossings (*n* = 15–19). **(I)** Mean velocity during the spatial probe test (*n* = 15–19). **(J)** The experimental protocol for FC. **(K)** The freezing time of rats during FC training. **(L)** The freezing time of rats in the context FC test (*n* = 15–19). **(M)** The freezing time of rats in the cued FC test (*n* = 15–19). LPS: lipopolysaccharide; NS: normal saline; MWM: Morris water maze; FC: fear conditioning; Panels B, D, and K were compared by two-way ANOVA with repeated measures followed by a Bonferroni post hoc test; Panel C was compared by log-rank test; Panels F, G, H, I, L, and M were compared by unpaired two-tailed Student’s t test; ^*^ *P* < 0.05, ^**^ *P* < 0.01, and ^***^ *P* < 0.001, n.s.: no significance; Error bars indicate SD

A neonatal inflammation model was induced in P3 rats (Fig. 1A). Compared with the control group, the body weight gain of rats was slower during development in the LPS group (Fig. 1B, *n*=8, ** *P* < 0.01, *** *P* < 0.001 vs. control group). No death was found in the rats from the control group, while a mortality of 36.7% was observed in the LPS group (Fig. 1C, ** P < 0.01). Training in the Morris water maze (MWM) task was performed from P28 to P31. During the 4 days of acquisition training, the escape latency decreased gradually in the rats of both the control and LPS groups, suggesting that both groups showed hippocampus-dependent spatial learning and memory formation (Fig. 1D). However, the escape latency in rats of the LPS group was significantly increased compared to control rats at training day 4, indicating a potential impairment in memory formation (Fig. 1D, *n*= 15–19, *P* = 0.029). During the spatial probe test (Fig. 1E), the time spent (Fig. 1F , *n*= 15–19, *** *P* < 0.001) and the distance (Fig. 1G, *n*= 15–19, *** *P* < 0.001) in the target quadrant, as well as the times crossing the platform (Fig. 1H , *n*= 15–19, *** *P* < 0.001), were decreased in the LPS group compared with the control group. No significant difference was found in the mean velocity of rats between the two groups (Fig. 1I , *n*= 15–19, *P* = 0.065), suggesting that the locomotor ability was not affected by LPS injection. The same rats were then subjected to a fear conditioning (FC) test (Fig. 1J). After training at P34 (Fig. 1K), rats in the LPS group exhibited decreased freezing in hippocampus-dependent contextual testing compared to that in the control group (Fig. 1L , *n*=15–19, *** *P* < 0.001), but no difference was observed for hippocampus-independent cue-tone testing at P35 (Fig. 1M , *n*= 15–19, *P* = 0.474).

Of note, additional analysis was performed during peer review to determine whether the neonatal severe inflammation-induced cognitive impairment was sex-dependent. No significant sex difference was found in MWM test as both male and female septic rats exhibited similar decrease in the time spent (Additional file 3*: *Fig. S1A left, *n* = 9, ** *P* < 0.01 for male; Additional file 3*: *Fig. S1A right, *n* = 6–10, ** *P* < 0.01 for female) and the distance (Additional file 3*: *Fig. S1B left, *n* = 9, ** *P* < 0.01 for male; Additional file 3*: *Fig. S1B right, *n* = 6–10, *P* = 0.046 for female) in the target quadrant, as well as the times crossing the platform (Additional file 3*: *Fig. S1C left, *n* = 9, *** *P* < 0.001 for male; Additional file 3*: *Fig. S1C right, *n* = 6–10, *** *P* < 0.001 for female) when compared to the control rats. Similarly, compared to control group, a decreased freezing in contextual testing (Additional file 3*: *Fig. S1D left*, n *= 9, *** *P* < 0.001 for male; Additional file 3*: *Fig. S1D right*, n* = 6–10, *** *P* < 0.001 for female) and no difference in cue-tone testing (Additional file 3*: *Fig. S1E left, *n* = 9, *P* = 0.301 for male; Additional file 3*: *Fig. S1E right, *n* = 6–10, *P* = 0.949 for female) were observed in both sexes of rats received LPS. These results suggest that neonatal severe inflammation can induce long-term cognitive impairments in both sexes.

### Neonatal severe inflammation induces sustained elevation of IL-1β in the rat hippocampus

**Fig. 2:**
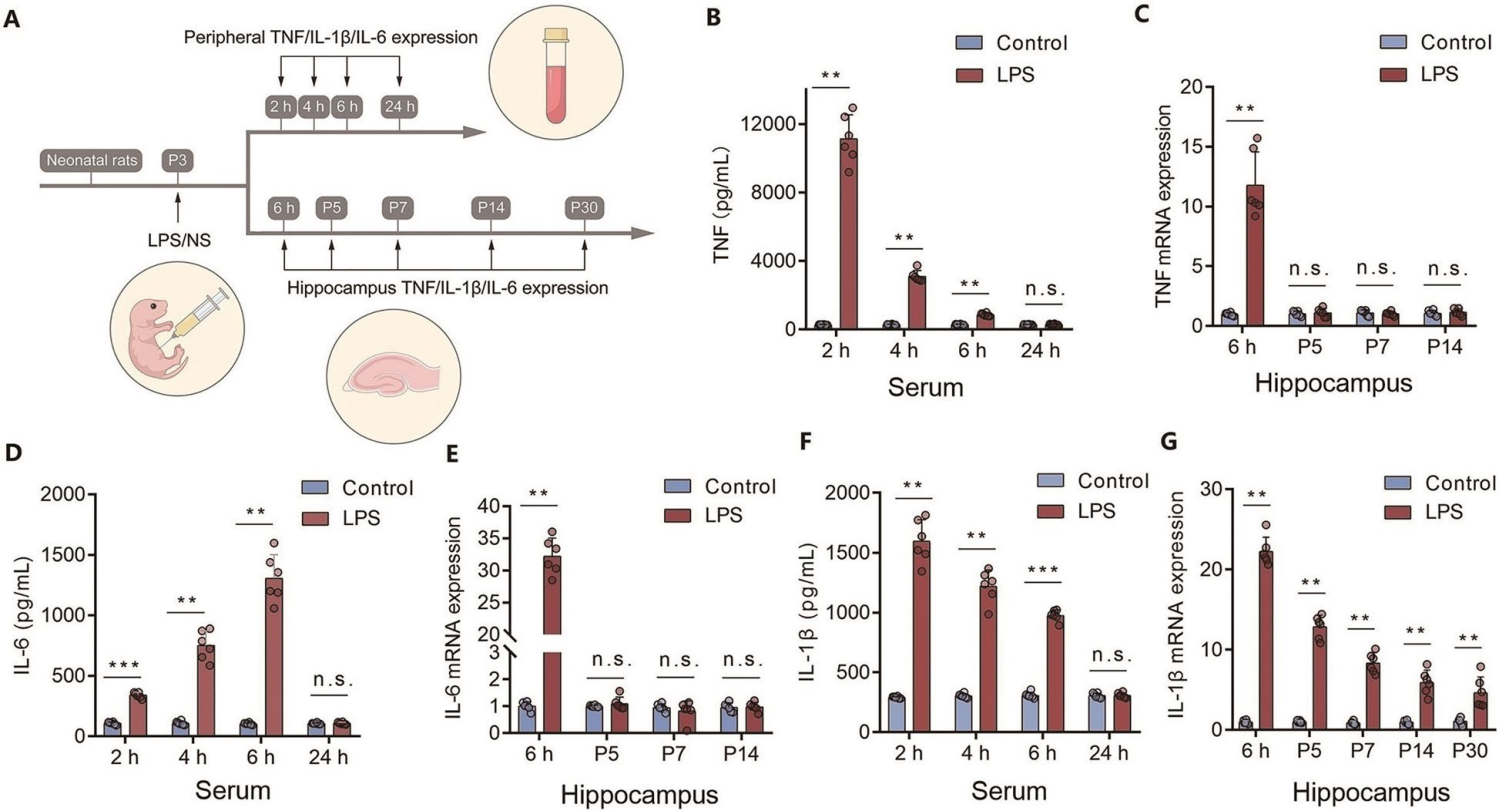
Neonatal severe inflammation induces sustained elevation of IL-1β in the rat hippocampus. **(A)** Schematic illustrating the chronological order used for the proinflammatory cytokine test after neonatal inflammation. Fourteen litters were used in this cohort of experiment. **(B, D, F)** ELISA results showing the protein levels of TNF (B), IL-6 (**D**), and IL-1β (**F**) in peripheral blood serum at 2 h (*n* = 6), 4 h (*n* = 6), 6 h (*n* = 6), and 24 h (*n* = 6). **(C, E, G)** PCR results showing the mRNA levels of hippocampal TNF (**C**) and IL-6 (**E**) at 6 h after LPS injection (*n* = 6), P5 (*n* = 6), P7 (*n* = 6), and P14 (*n* = 6), and IL-1β after LPS injection (**G**) at 6 h (*n* = 6), P5 (*n* = 6), P7 (*n* = 6), P14 (*n* = 6), and P30 (*n* = 6). LPS: Lipopolysaccharide, NS: Normal saline, Panels B, C, D, E, F, and G were compared by unpaired two-tailed Student’s t test or Mann–Whitney U test; ^**^ *P* < 0.01 and ^***^ *P* < 0.001, n.s.: no significance; Error bars indicate SD

The levels of proinflammatory cytokines in peripheral blood were examined by ELISA at different timepoints after LPS injection (Fig. 2A). The results showed that TNF (Fig. 2B, *n*= 6, ** *P* < 0.01), IL-6 (Fig. 2D, *n*= 6, ** *P* < 0.01, *** *P* < 0.001) and IL-1β (Fig. 2F*, n* = 6, ** *P* < 0.01, *** *P* < 0.001) in peripheral blood serum were significantly increased at 2 h, 4 h, and 6 h after LPS injection, but all returned to control levels at 24 h after LPS injection (Fig. 2B, 2D, 2F, *n*=6, *P* > 0.05). Then, we examined the expression levels of these proinflammatory cytokines in the hippocampus by RT-PCR and Western blotting. Compared to the control rats, the mRNA expression levels of TNF (Fig. 2C, *n*= 6, ** *P* < 0.01) and IL-6 (Fig. 2E, *n*= 6, ** *P* < 0.01) were markedly elevated in the hippocampus at 6 h post-injection of LPS, but both returned to control levels at P5 (Fig. 2C*, *2E, *n*= 6, *P* > 0.05). Notably, the elevated levels of IL-1β mRNA (Fig. 2G, *n*= 6, ** *P* < 0.01) and protein (Additional file 3: Fig. S2*,* *n* = 6, ** *P* < 0.01, *** *P* < 0.001) were maintained at least until P30, suggesting that IL-1β was the predominant proinflammatory cytokine in the CNS after severe neonatal inflammation.

### Sustained elevation of hippocampal IL-1β levels contributes to long-term cognitive impairment after neonatal severe inflammation

**Fig. 3:**
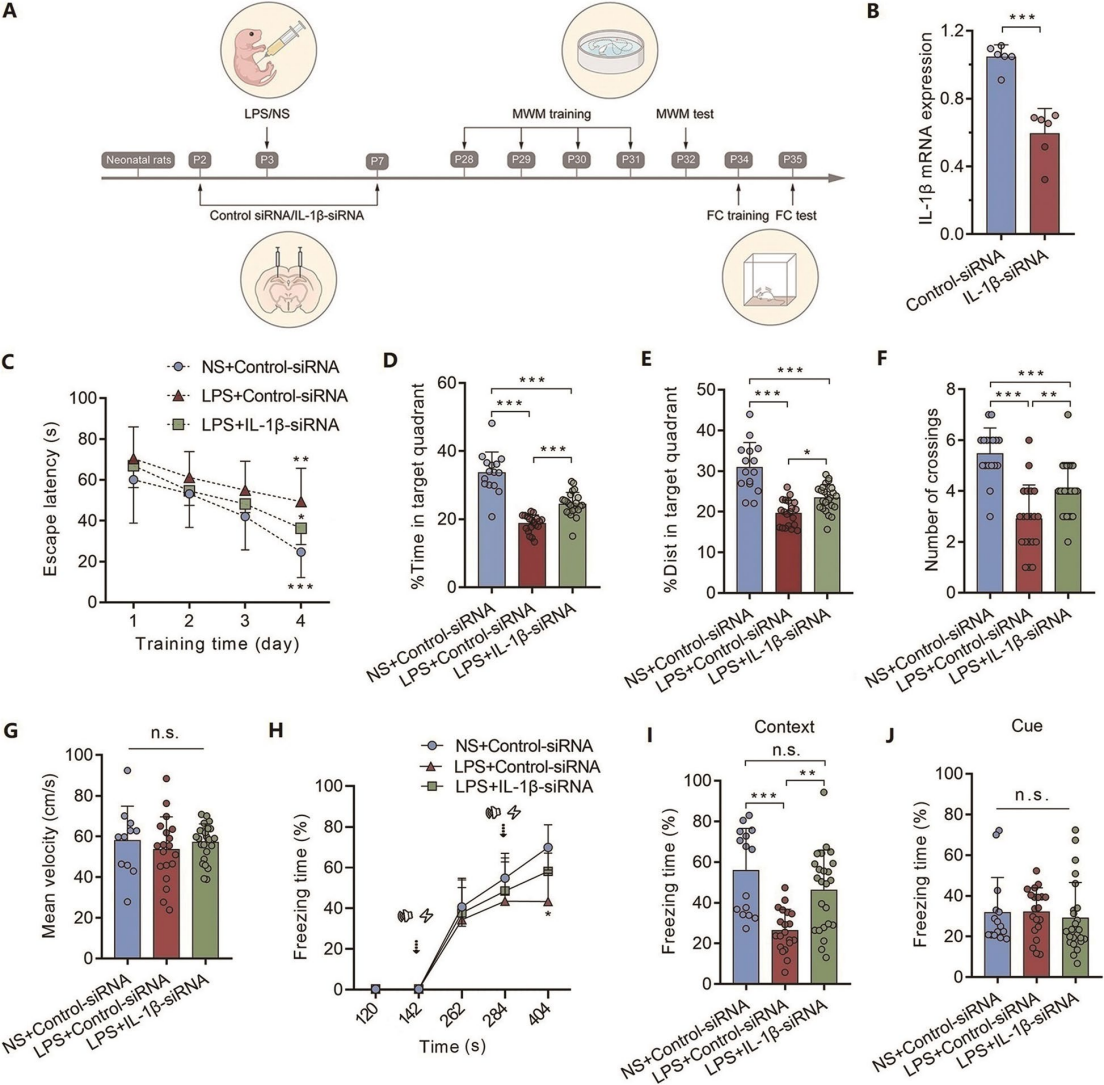
Sustained elevation of hippocampal IL-1β levels contributes to long-term cognitive impairment after neonatal severe inflammation. **(A)** Schematic illustrating the chronological order used for siRNA delivery, LPS administration and cognitive testing. Eight litters were used in this cohort of experiment. **(B)** PCR results showing the knockdown efficiency of IL-1β-siRNA (*n* = 6). **(C)** Learning curve for the escape latency. **(D)** Time spent in the target quadrant (*n* = 15–24). **(E)** Distance spent in the target quadrant (*n* = 15–24). **(F)** Number of platform crossings (*n* = 15–24). **(G)** Mean velocity during the spatial probe test (*n* = 15–24). **(H)** The freezing time of rats in FC training. **(I)** The freezing time of rats in the context FC test (*n* = 15–24). **(J)** The freezing time of rats in the cued FC test (*n* = 15–24). LPS: lipopolysaccharide; NS: normal saline; MWM: Morris water maze; FC: fear conditioning; Panel B was compared by unpaired two-tailed Student’s t test; Panels C and H were compared by two-way ANOVA with repeated measures followed by a Bonferroni post hoc test; Panels D, E, F, G, I, and J were compared by one-way ANOVA with repeated measures followed by a Tukey post hoc test; ^**^ *P* < 0.01 and ^***^ *P* < 0.001, n.s.: no significance; Error bars indicate SD

To determine the role of the sustained increase in IL-1β levels in neonatal inflammation-induced cognitive impairment, IL-1β-siRNA was bilaterally injected into the CA1 region of the hippocampus to knockdown the expression of IL-1β mRNA (Fig. 3A). The fluorescence carried by IL-1β-siRNA was detected in CA1 (Additional file 3: Fig. S3A). The IL-1β mRNA levels were significantly decreased by IL-1β-siRNA two days after injection (Fig. 3B, *n*= 6, *** *P* < 0.001). Compared to the LPS + control-siRNA group, the escape latency in rats from the NS + control-siRNA (Fig. 3C, *** *P* < 0.001) and LPS + IL-1β-siRNA (Fig. 3C, ** *P* < 0.01) groups was significantly decreased at training day 4 of MWM. For MWM test, treatment with IL-1β-siRNA significantly improved neonatal inflammation-induced cognitive impairment (Fig. 3D-3F, *n*= 15–24, *P = 0.012, ** *P* < 0.01, *** *P* < 0.001). No significant difference was found in the mean velocity of rats among all three groups (Fig. 3G, *n*= 15–24, *P* = 0.611). For FC, after training (Fig. 3H), rats in the LPS + IL-1β-siRNA group exhibited increased freezing in hippocampus-dependent contextual testing compared to that in the LPS + control-siRNA group (Fig. 3I, *n*= 15–24, ** *P* < 0.01, *** *P* < 0.001). No difference was observed for hippocampus-independent cue-tone testing among these three groups (Fig. 3J, *n*= 15–24, *P* = 0.786). These findings indicate that sustained elevated levels of IL-1β contribute to long-term cognitive impairment after neonatal inflammation.

### Neonatal severe inflammation upregulates expression of KCC2 during development

**Fig. 4:**
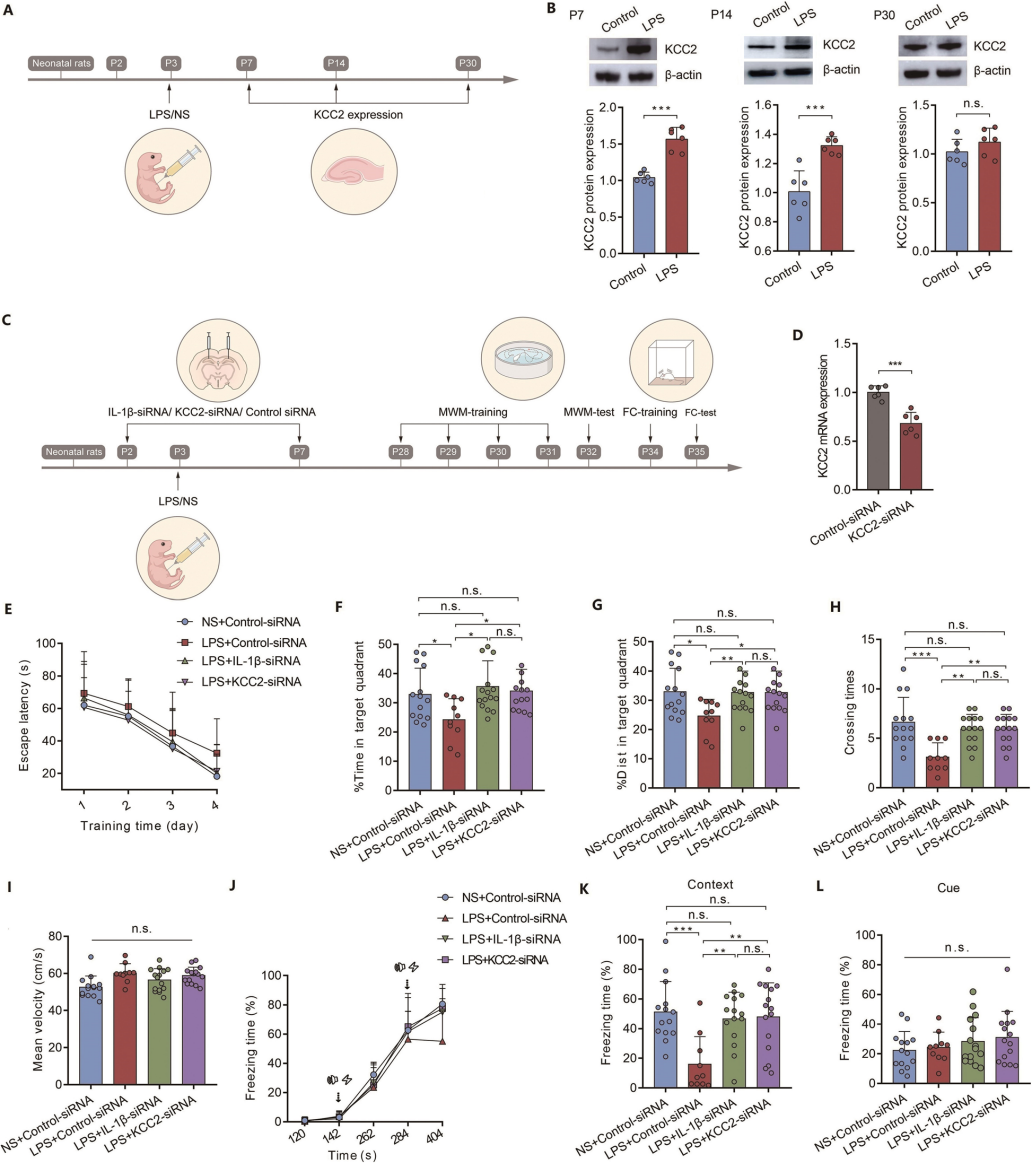
KCC2 mediates the effects of IL-1β on neonatal severe inflammation-induced cognitive impairment. **(A)** Schematic illustrating the chronological order used for the establishment of the inflammation model and KCC2 level testing. Five litters were used in this cohort of experiment. **(B)** The protein levels of KCC2 in P7 (left panel, *n* = 6), P14 (middle panel, *n* = 6), and P30 (right panel, *n* = 6) rats after LPS injection. **(C)** Schematic illustrating the chronological order used for siRNA injection, establishment of the inflammation model, and cognitive testing. Nine litters were used in this cohort of experiment. **(D)** The knockdown efficiency of KCC2-siRNA by PCR (*n* = 6). **(E)** Learning curve for the escape latency. **(F)** Time spent in the target quadrant (*n* = 10–15). **(G)** Distance spent in the target quadrant (*n* = 10–15). **(H)** Number of platform crossings (*n* = 10–15). **(I)** Mean velocity during the spatial probe test (*n* = 10–15). **(J)** The freezing time of rats during FC training. **(K)** The freezing time of rats in the context FC test (*n* = 10–15). **(L)** The freezing time of rats in the cued FC test (*n* = 10–15). LPS: lipopolysaccharide; NS: normal saline; MWM: Morris water maze; FC: fear conditioning; Panels B and D were compared by unpaired two-tailed Student’s t test; Panels F, G, H, I, K and L were compared by one-way ANOVA with repeated measures followed by a Tukey post hoc test; ^*^ *P* < 0.05, ^**^ *P* < 0.01, and ^***^ *P* < 0.001, n.s.: no significance; Error bars indicate SD

Firstly, we determined whether KCC2 expression were sex-specific during development in rats. KCC2 expression levels gradually increased in the hippocampus from P3 to P14. The expression levels of KCC2 (Additional file 3: Fig. S4A, *n* = 6, *P* > 0.05; Additional file 3: Fig. S4B, *n* = 4, *P* > 0.05) showed no sex difference from P3 to P14. Then, we found that LPS injection at P3 induced an increased expression of KCC2 at P7 (Fig. 4B left panel*,* *n* = 6, *** *P* < 0.001) and P14 (Fig. 4B middle panel*,* *n* = 6, *** *P* < 0.001) compared to the normal control. No difference was found in KCC2 (Fig. 4B right panel*,* *n* = 6, *P* > 0.05) expression in rats at P30 between the LPS group and the control group. These results indicate that neonatal inflammation may accelerate increased expression level of KCC2 during early development.

### KCC2 mediates the effects of IL-1β on the long-term cognitive impairment induced by neonatal severe inflammation

KCC2-siRNA was used to decrease the expression of KCC2 in CA1 of the hippocampus. The fluorescence carried by KCC2-siRNA was detected in CA1 (Additional file 3: Fig. S3B). The knockdown efficiency of KCC2-siRNA was confirmed by RT-PCR (Fig. 4D, *n*= 6, *** *P* < 0.001). IL-1β-siRNA injection decreased the expression of IL-1β in the hippocampus at P7 (Additional file 3: Fig. S5A left panel, *n* = 6–8, *** *P* < 0.001; Additional file 3: Fig. S6 left panel, *n* = 6, *** *P* < 0.001), P14 (Additional file 3: Fig. S5A middle panel, *n* = 6, ** *P* < 0.01; Additional file 3: Fig. S6 middle panel, *n* = 6, ** *P* < 0.01) and P30 (Additional file 3: Fig. S5A right panel, *n* = 6, * *P* = 0.037; Additional file 3: Fig. S6 right panel, *n* = 6, * *P* = 0.013) after LPS injection at P3. KCC2-siRNA injection decreased the expression of KCC2 in the hippocampus at P7 (Additional file 3: Fig. S5B left panel, *n* = 6, * *P* = 0.025; Additional file 3: Fig. S7 left panel, *n* = 6, * *P* = 0.024) and P14 (Additional file 3: Fig. S5B middle panel, *n* = 6, ** *P* < 0.01; Additional file 3: Fig. S7 middle panel, *n* = 6, * *P* = 0.014) after LPS injection at P3. Moreover, knockdown of IL-1β expression inhibited the increase in KCC2 expression (Additional file 3: Fig. S5B left and middle panel, *n* = 6, * *P* = 0.044 for P7, * *P* = 0.038 for P14; Additional file 3: Fig. S7 middle panel, *n* = 6, * *P* = 0.037 for P14) induced by LPS injection. Accordingly, the cognitive impairment induced by neonatal severe inflammation was significantly improved by IL-1β-siRNA and/or KCC2-siRNA injection (Fig. 4F-4H, 4K, *n*= 10–15, * *P* < 0.05, ** *P* < 0.01, *** *P* < 0.001), indicating that inhibiting the elevation of IL-1β and/or KCC2 expression during the period of GABAergic development was able to prevent the long-term cognitive impairment that induced by neonatal severe inflammation.

### Neonatal severe inflammation accelerates GABAergic shift during development

**Fig. 5:**
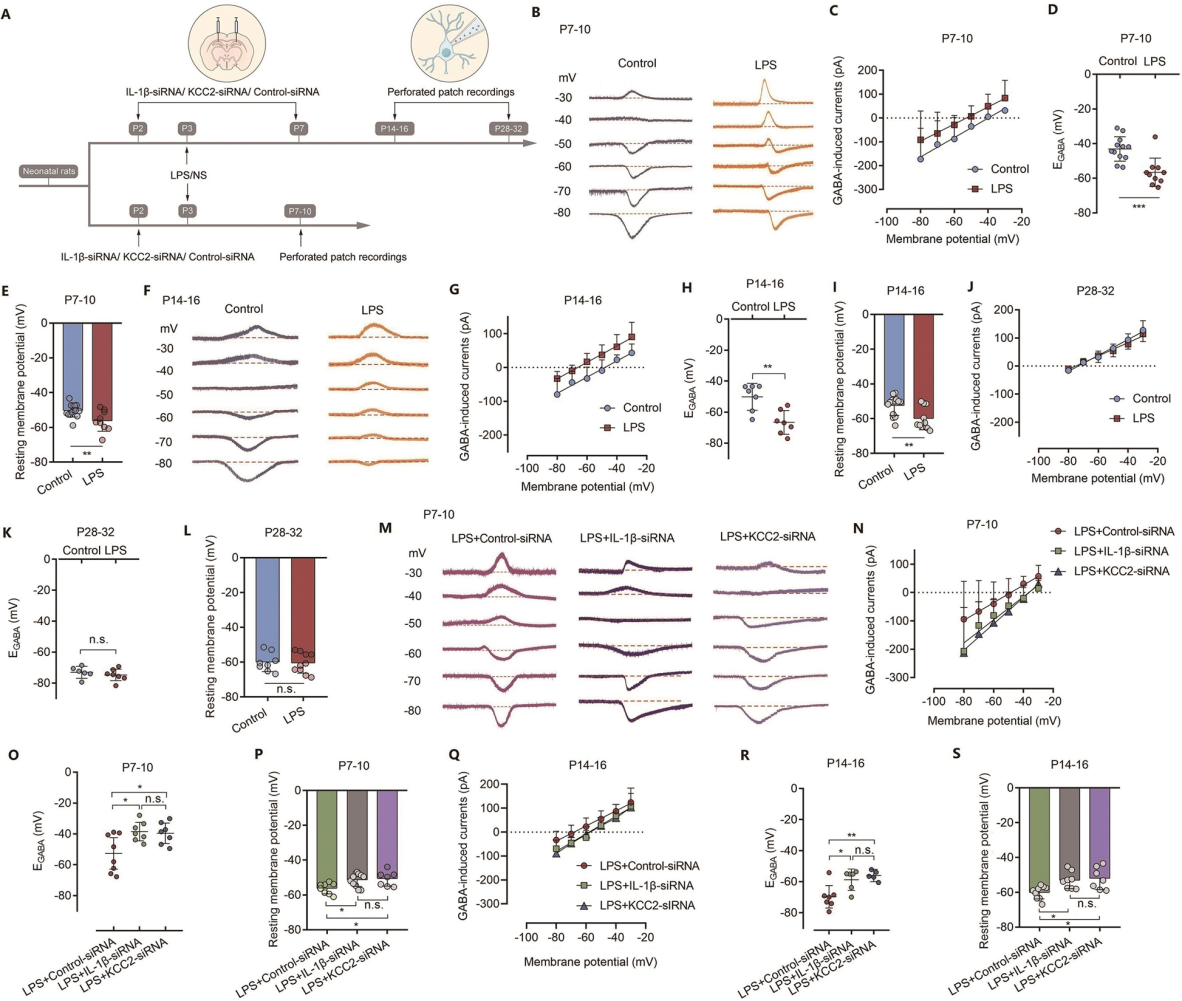
E_GABA_ is hyperpolarized in CA1 pyramidal neurons of rats after neonatal severe inflammation. **(A)** Schematic illustrating the chronological order used for siRNA delivery, LPS administration and perforated patch recordings. Fifteen litters were used in this cohort of experiment. **(B)** Representative traces of GABA-induced currents to the holding potential from –80 to –30 mV in 10 mV increments of hippocampal neurons at P7-P10. **(C)** Current–voltage (I-V) curve of GABA-induced currents recorded at different holding potentials from − 80 to − 30 mV in 10 mV increments of pyramidal neurons at P7-P10. **(D)** E_GABA_ values per cell obtained from all I-V curves indicating a hyperpolarizing shift in septic rats at P7-P10 (*n* = 10–12 cells from 4–5 rats). **(E)** RMP values showing a hyperpolarizing shift in septic rats at P7-10 (*n* = 9–14 cells from 5–6 rats). **(F)** Representative traces of GABA-induced currents to the holding potential from –80 to –30 mV in 10 mV increments of hippocampal neurons at P14-P16. **(G)** Current–voltage (I-V) curve of GABA-induced currents recorded at different holding potentials from − 80 to − 30 mV in 10 mV increments of pyramidal neurons at P14. **(H)** E_GABA_ values per cell obtained from all I-V curves indicating a hyperpolarizing shift in septic rats at P14-P16 (*n* = 7 cells from 3–4 rats). **(I)** RMP values showing a hyperpolarizing shift in septic rats at P14-P16 (*n* = 10–13 cells from 4–5 rats). **(J)** Current–voltage (I-V) curve of spontaneous GABA-induced currents recorded at different holding potentials from − 80 to − 30 mV in 10 mV increments of pyramidal neurons at P28-P32. **(K)** E_GABA_ values per cell obtained from all I-V curves indicating a hyperpolarizing shift in septic rats at P28-32 (*n* = 6–7 cells from 3–4 rats). **(L)** RMP values showing a hyperpolarizing shift in septic rats at P28-P32 (*n* = 8–10 cells from 4–5 rats). **(M)** Representative traces of GABA-induced currents to the holding potential from − 80 to − 30 mV in 10 mV increments of hippocampal neurons at P7-P10 after siRNA injection. **(N)** Current–voltage (I-V) curve of GABA-induced currents recorded at different holding potentials from − 80 to − 30 mV in 10 mV increments of pyramidal neurons at P7-P10 after siRNA injection. **(O)** E_GABA_ values per cell obtained from all I-V curves at P7-P10 after siRNA injection (n = 7–8 cells from 3–4 rats). **(P)** RMP values at P7-P10 after siRNA injection (*n* = 7–11 cells from 4–5 rats). **(Q)** Current–voltage (I-V) curve of GABA-induced currents recorded at different holding potentials from − 80 to − 30 mV in 10 mV increments of pyramidal neurons at P14-P16 after siRNA injection. **(R)** E_GABA_ values per cell obtained from all I-V curves at P14-P16 after siRNA injection (*n* = 5–7 cells from 3–4 rats). **(S)** RMP values at P14-P16 after siRNA injection (*n* = 7–9 cells from 3–4 rats). LPS: lipopolysaccharide; NS: normal saline; E_GABA_: GABA reversal potential; Panels D, E, H, I, K, and L were compared by unpaired two-tailed Student’s t test; Panels O, P, R, and S were compared by one-way ANOVA with repeated measures followed by a Tukey post hoc test; ^*^ *P* < 0.05, ^**^ *P* < 0.01, and ^***^ *P* < 0.001, n.s.: no significance; Error bars indicate SD

Next, we addressed the effects of neonatal inflammation on the intrinsic electrophysiological properties of the CA1 pyramidal neurons of rats at P7-P10, P14-P16 and P28-P32 (Fig. 5A). The GABA reversal potential (E_GABA_) was determined to directly test the changes in chloride homeostasis of CA1 slices following neonatal severe inflammation. Neonatal inflammation caused a significant hyperpolarizing shift in E_GABA_ at both P7-P10 (Fig. 5B-5D, *n*= 10–12 cells from 4–5 rats, *** *P* < 0.001) and P14-P16 (Fig. 5F-5H, *n*= 7 cells from 3–4 rats, ** *P* < 0.01), accompanied by a hyperpolarized resting membrane potential (RMP) (Fig. 5E, *n*= 9–14 cells from 5–6 rats, ** *P* < 0.01; Fig. 5I, *n*= 10–13 cells from 4–5 rats, ** *P* < 0.01). Knockdown of IL-1β expression or KCC2 expression alleviated the neonatal inflammation-induced hyperpolarizing shift in E_GABA_ (Fig. 5M-5O, *n*= 7–8 cells from 3–4 rats, * *P* = 0.02 vs. LPS + IL-1β-siRNA group, * *P *= 0.033 vs. LPS + KCC2-siRNA group; Fig. 5Q-5R, *n*= 5–7 cells from 3–4 rats, * *P* = 0.02, ** *P* < 0.01) and hyperpolarized RMP (Fig. 5P, *n*= 7–11 cells from 4–5 rats, * *P* = 0.025 vs. LPS + IL-1β-siRNA group, * *P* = 0.023 vs. LPS + KCC2-siRNA group; Fig. 5S, *n*= 7–9 cells from 3–4 rats, * *P *= 0.022 vs. LPS + IL-1β-siRNA group, * *P* = 0.011 vs. LPS + KCC2-siRNA group) at both P7-P10 and P14-P16. No significant difference was found in the E_GABA_ (Fig. 5J-5K, *n*= 6–7 cells from 3–4 rats, P > 0.05) or the RMP (Fig. 5L, *n*= 8–10 cells from 4–5 rats, *P* > 0.05) in P28-P32 rats between the LPS and control groups.

## Discussion

The present study reveals that neonatal severe inflammation can induce long-lasting cognitive impairment in adolescent rats via upregulation of IL-1β/KCC2 signaling during neonatal development, accompanied by accelerating GABAergic shift from depolarizing to hyperpolarizing.

It is generally recognized that CNS inflammation plays a critical role in the development of long-lasting cognitive impairment following early life inflammation [35, 36]. Proinflammatory cytokines, particularly IL-1β, play an important role in the CNS inflammation process after neonatal inflammation [16, 17]. Moreover, IL-1β is well known to influence hippocampus-dependent memories and learning [37]. Consistent with previous evidence [10], our results showed that IL-1β, but not IL-6 and TNF, was sustained at a high level at least until postnatal day 30 after LPS injection at P3. Notably, knockdown of the expression of IL-1β significantly alleviated the long-lasting cognitive impairment induced by neonatal inflammation, confirming the important role of sustained elevated levels of IL-1β in this disorder.

GABA depolarizes immature neurons during early postnatal days [38, 39]. During neuronal maturation, there is a GABA-mediated functional shift from depolarizing to hyperpolarizing by upregulation of the chloride exporter KCC2, leading to a negative shift in the reversal potential for chloride ions [20, 38, 40]. Insults during such developmental time windows may induce long-term consequences [27, 40]. Here we proposed that neonatal inflammation may alter the expression of KCC2, thus affecting the GABAergic shift during development, which may contribute to long-lasting cognitive impairment. As expected, our results demonstrated that neonatal inflammation increased the expression of KCC2, thus maintaining a lower concentration of intracellular Cl^–^, as evidenced by a hyperpolarized E_GABA_. Notably, knockdown of KCC2 expression alleviated the cognitive impairment induced by neonatal inflammation and reversed hyperpolarized E_GABA_. To determine whether KCC2 is a downstream target of IL-1β, we examined KCC2 expression and E_GABA_ after IL-1β-siRNA injection in LPS rats. As a result, knockdown of the expression of IL-1β can reverse the changed expression of KCC2 and E_GABA_. Therefore, our findings indicate that the upregulation of KCC2 during development mediated the effects of elevated IL-1β levels on long-lasting cognitive impairment. Whereas, Corradini et al. reported that maternal infection with polyinosinic-polycytidylic acid (PolyI:C) causes downregulation of KCC2 transcription in the cortex of offspring mice, thus leading to delayed excitatory-to-inhibitory GABAergic shift and higher susceptibility to seizures in vivo, which endures up to adulthood [27]. These abnormities were not observed in Interleukin-1 receptor type I knockout mice [27]. Their findings appear to be contrary to our results, which may result from the different brain regions and time window of inflammation. Previous studies have confirmed that the function of GABAergic transmission was region-dependent, such as cortex vs. hippocampus [18–20]. In addition, the higher dose of LPS used in the present study maybe also a contributor for such discrepancy. In summary, both their findings and our results here suggested a link between IL-1β/KCC2 and GABAergic shift during development; and confirmed that the abnormal GABAergic shift, either acceleration or delay, may lead to neurodevelopmental defects.

Gomez et al. found that early-life inflammation increases CA1 pyramidal neuron excitability in adult male mice, as demonstrated by a depolarized GABA reversal potential resulting from an increased expression of NKCC1 [13]. Therefore, both their findings and our results highlight the role of chloride homeostasis in the long-lasting intrinsic membrane properties in hippocampal neurons after early-life inflammation, although some discrepancies exist. In our present study, we did not observe a significant sex difference in neonatal severe inflammation-induced long-term cognitive impairment. Moreover, our results showed that upregulation of KCC2 plays the causal role. The timepoints of LPS administration may be the major cause for the discrepancy: neonatal inflammation was induced by LPS injection at postnatal day 14 (P14) in Gomez’s study [13], whereas the LPS was injected at P3 in this present study. In the study of Gomez et al. [13], patch recordings from CA1 hippocampal pyramidal neurons were performed at adolescence (P35-P45) or adulthood (P60-P70) and showed a depolarized E_GABA_. While in this study, patch recordings were recorded at P7-P10 and/or P14-P16 and showed a hyperpolarized E_GABA_. Previous evidence showed that the GABAergic shift may have been almost finished after P14 [20]; therefore, inflammation induced at early stage vs. almost complete stage of GABAergic shift may lead to different results. In addition, inflammation at older age near adolescent may prone to cause sex-dependent cognitive disorders. Of note, as above mentioned, the higher dose of LPS used in the present study maybe also a contributor for such discrepancy. Unlike to the dose of LPS (0.1 mg kg^−1^, *i.p.*) in Gomez’s study [13] and another study [41], we used a higher dose of LPS (1 mg kg^−1^, *i.p.*) which resulted to ~ 40% mortality. Therefore, such high dose of LPS resembles a sepsis model rather than a neonatal inflammation. Importantly, we performed behavioural experiments and demonstrated that upregulation of KCC2 and accelerated GABAergic shift is an important contributor to cognitive impairments induced by neonatal inflammation. A limitation is that we did not test whether a similar effect was seen in adult rats as reported by Gomez and colleagues [13]. Future studies are needed to explore whether the effect of neonatal inflammation is limited to a specific time window.

Besides KCC2, NKCC1 also plays a pivotal role in the neuronal development of immature brain [18, 42]. NKCC1 has been suggested to be an important therapeutic target for various neurodegenerative diseases. For example, cognitive impairment in in a murine model of schizophrenia was associated with the reversal potential of GABA_A_ currents in pyramidal neurons of the infralimbic prefrontal cortex that resulted from an increased expression of NKCC1, which could be improved by bumetanide [43]. In a mouse model of Down syndrome, NKCC1 knockdown in vivo rescues cognitive deficits in diverse behavioural tasks [44]. Treatment with bumetanide, a NKCC1 antagonist, during a vulnerable developmental period rescues epilepsy in a genetic epilepsy mice model [45]. Furthermore, Gomez and colleagues have confirmed the role of NKCC1 in early life inflammation-induced intrinsic membrane properties in hippocampal neurons [13]. However, one study revealed that NKCC1 in telencephalic glutamatergic neurons appears to not be essential for major aspects of hippocampal development [46]. In future studies, it will be interesting to determine the role of NKCC1 in neonatal inflammation-induced cognitive impairment.

In the present study, rats were tested for both hippocampus-dependent contextual and hippocampus-independent cued fear conditioning [47]. Neonatal severe inflammation affected hippocampus-dependent contextual but not cued fear conditioning. These data, together with the spatial learning and memory outcomes detected by the hippocampus-dependent Morris water maze cognitive tests, highlight the importance of the hippocampus in neonatal severe inflammation-induced long-lasting cognitive impairment.

The expression of KCC2 and E_GABA_ returned to the control levels in adolescent rats after neonatal inflammation, raising a question that what is the direct cause of long-term cognitive dysfunction at the time of behavioural measurement? Although the present study did not propose direct data, it is possible that KCC2 has multifaceted modulatory roles in neural development that related to cognitive functions, mainly including setting the strength and polarity of GABA currents during neuronal maturation, regulating cytoskeletal dynamics via its C-terminal domain, modulating developmental apoptosis, controlling the early network events, as well as implicating in the formation and plasticity of cortical dendritic spines [18]. Therefore, any abnormity in either above-mentioned functions may contribute to the neonatal inflammation-induced long-term cognitive defects. For example, it is possible that early-life inflammation and/or GABAergic shift influences the development of excitatory glutamatergic transmission [20, 48], thus leading to an impaired glutamatergic function in adolescence. Future studies are required to investigate why upregulation of KCC2 in early development period cause long-term cognitive impairment in adolescence or even adult.

It should be noticed that there are well-known effects of transport for pregnant dams [49, 50]. Therefore, transport of pregnant dams is not recommended for developmental studies and should be avoided in future experiments. In this study, although both controls and dams of inflamed pups were equally exposed to transport, there is a possibility that an interaction of the transport stress and the LPS-induced inflammation may exist.

## Conclusions

In summary, our results highlight a mechanistic link between the expression of IL-1β/KCC2 and long-lasting cognitive impairment in a neonatal severe inflammation model and provide an underlying molecular target to prevent and/or treat cognitive disorders after early septic inflammation.

## Supplementary Information

**Additional file 1. **Original images for Western blotting results. 

**Additional file 2: Table S1-S11. **Table. S1: Statistical information for Fig. 1. Table. S2: Statistical information for Fig. 2. Table. S3: Statistical information for Fig. 3. Table. S4: Statistical information for Fig. 4. Table. S5: Statistical information for Fig. 5. Table. S6: Statistical information for Supplementary figure 1. Table. S7: Statistical information for Supplementary figure 2. Table. S8: Statistical information for Supplementary figure 4. Table. S9: Statistical information for Supplementary figure 5. Table. S10: Statistical information for Supplementary figure 6. Table. S11: Statistical information for Supplementary figure 7. 

**Additional file 3: Fig. S1-S7. **Fig. S1: MWM task and FC test for male and female rats after neonatal inflammation. Fig. S2: Western blotting results showing the protein levels of hippocampal IL-1β at P7, P14, and P30 after LPS exposure. Fig. S3: Representative images showing the fluorescence carried by IL-1β-siRNA or KCC2-siRNA. Fig. S4: The mRNA and protein levels of hippocampal KCC2 with development in rats of both sexes. Fig. S5: The mRNA levels of hippocampal IL-1β and KCC2 in P7, P14, and P30 rats after siRNA injection. Fig. S6: The protein levels of hippocampal IL-1β in P7, P14, and P30 rats after siRNA injection. Fig. S7: The protein levels of hippocampal KCC2 in P7, P14, and P30 rats after siRNA injection. 

## Data Availability

The datasets used and/or analysed during the current study are available from the corresponding author on reasonable request.

## Abbreviations

CNS: Central nervous system
E_GABA_: GABA reversal potential
ELISA: Enzyme-linked immunosorbent assay
FC: Fear conditioning
GABA: γ-Aminobutyric acid
IL-1β: Interleukin-1 beta
KCC2: K^+^-Cl– co-transporter 2
LPS: Lipopolysaccharide
MWM: Morris water maze
NKCC1: Na^+^-K^+^-2Cl^–^ co-transporter 1
NS: Normal saline
P3: Postnatal day 3
RMP: Resting membrane potential
