# Supplementary material for: Severe inflammation in new-borns induces long-term cognitive impairment by activation of IL-1β/KCC2 signaling during early development
Source: BMC Med. 2022 Jul 27;20:235. doi: 10.1186/s12916-022-02434-w (PMC9327322; doi:10.1186/s12916-022-02434-w)

Notes:

**a.** All the gel images in this file are original uncropped blots images after exposure. To save antibody, we cut the gels according to makers before exposure and incubated with primary and secondary antibodies respectively.

**b.** There are some lines of blots maybe not presented in the results of this study (e.g., Minocyline group and Bumetanide group); because they were removed during peer review.

**c.** The name of blots in the red square are the representative blots that shown in main body of manuscript.


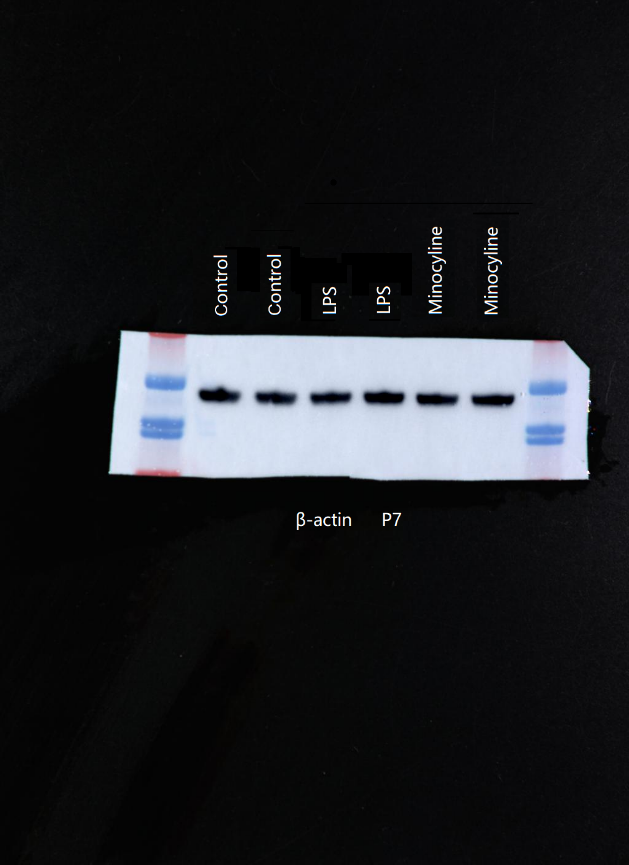
Original gels for Fig. 4B left panel.


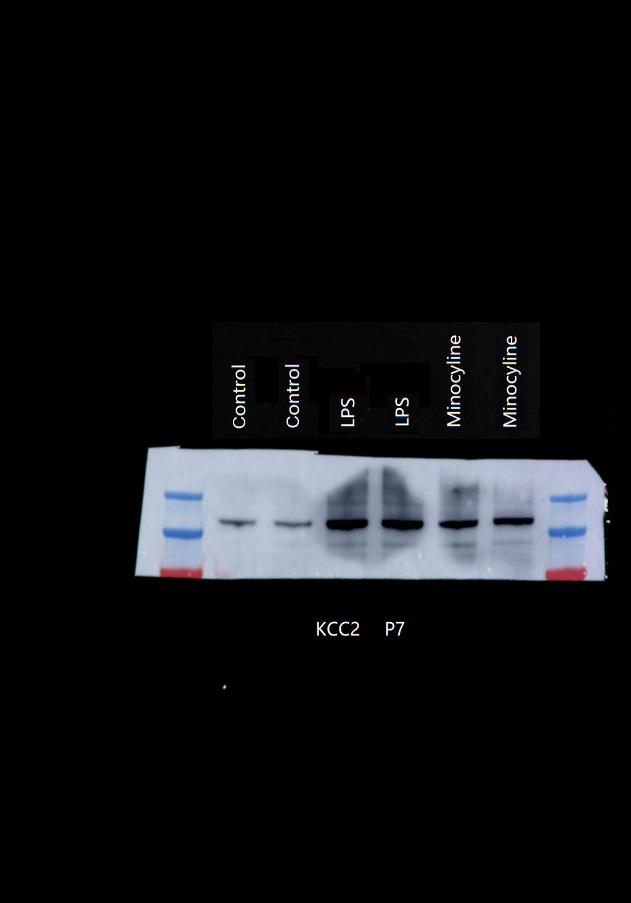


Original gels for Fig. 4B middle panel.


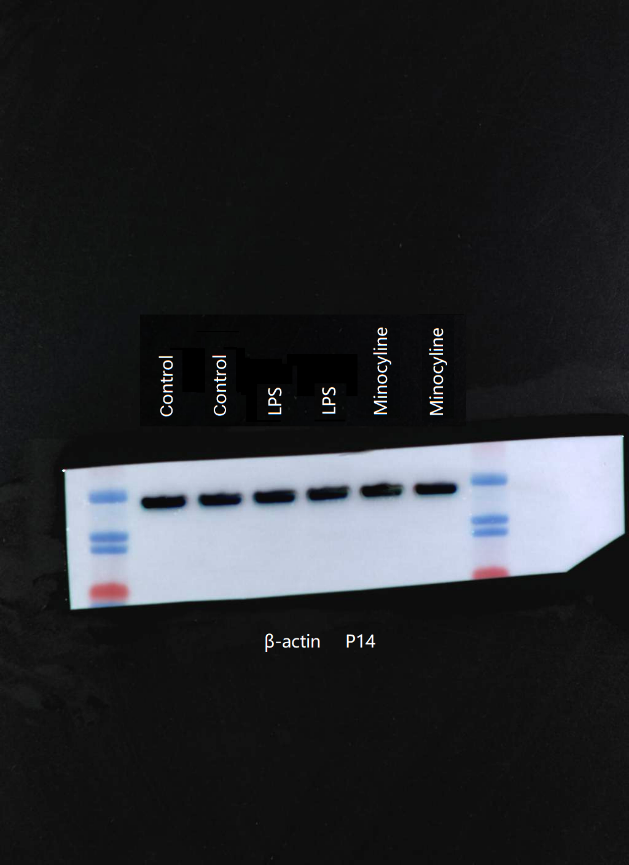

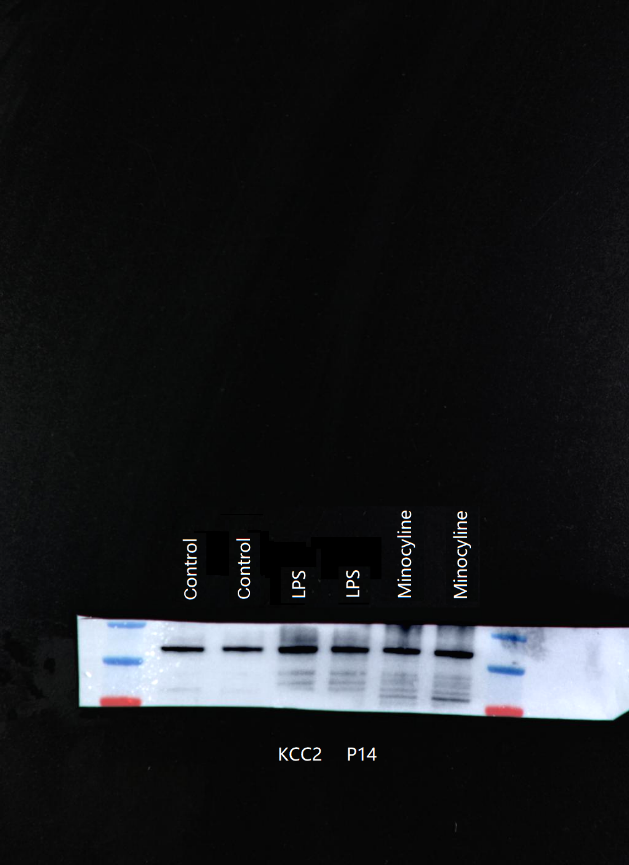


Original gels for Fig. 4B right panel.


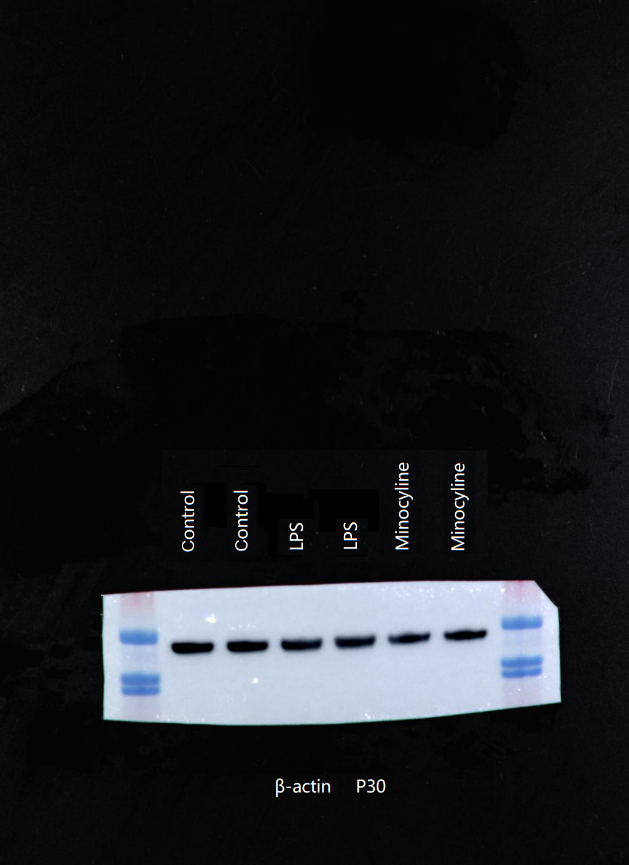

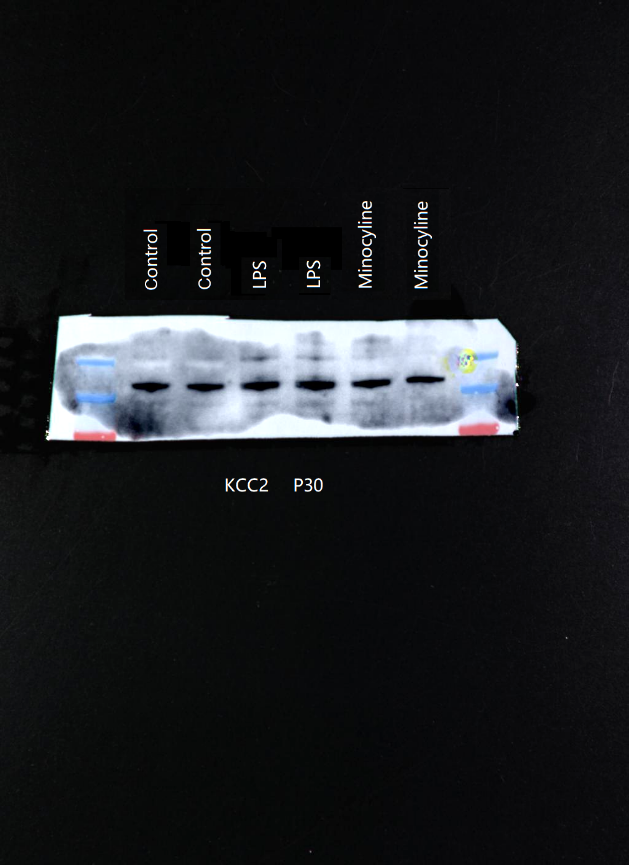


Original gels for Fig. S2 left panel.


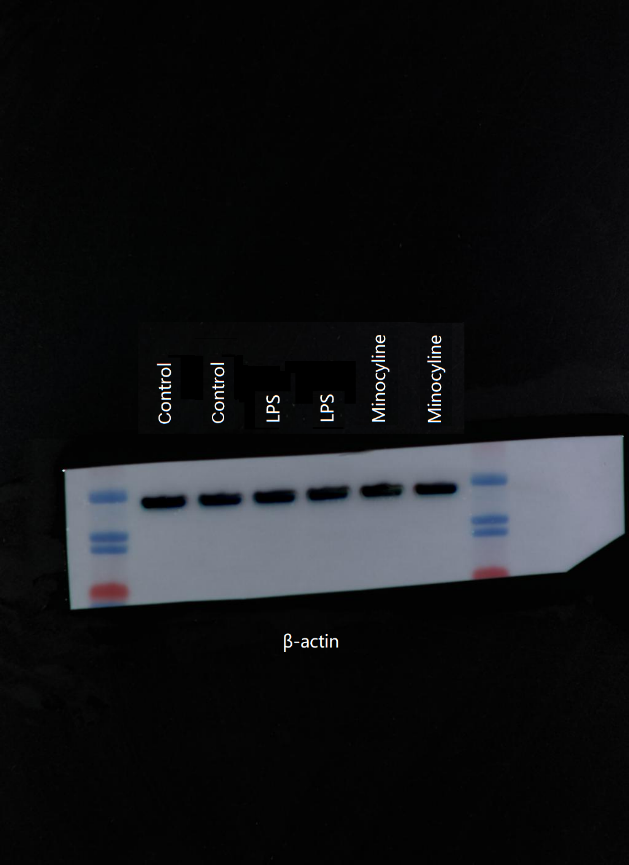

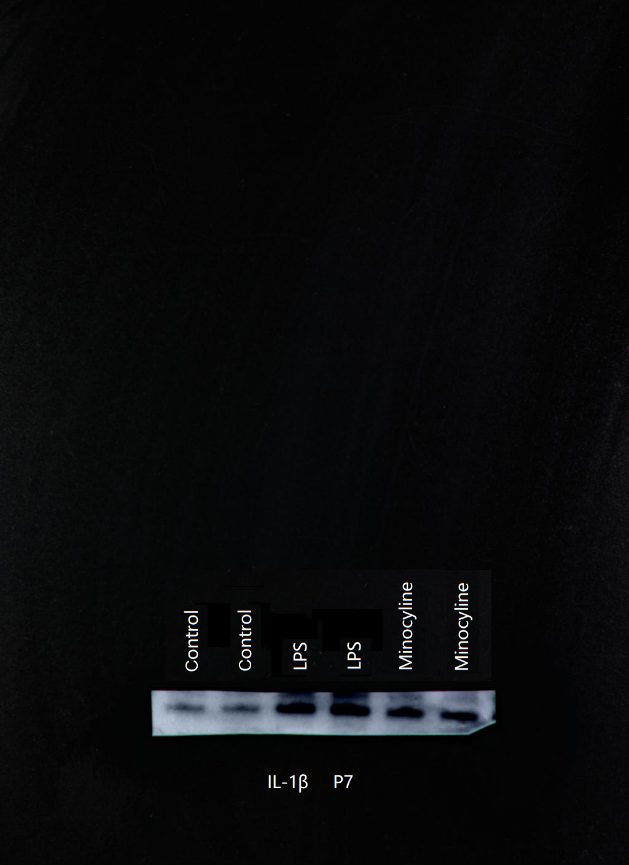


Original gels for Fig. S2 middle panel.


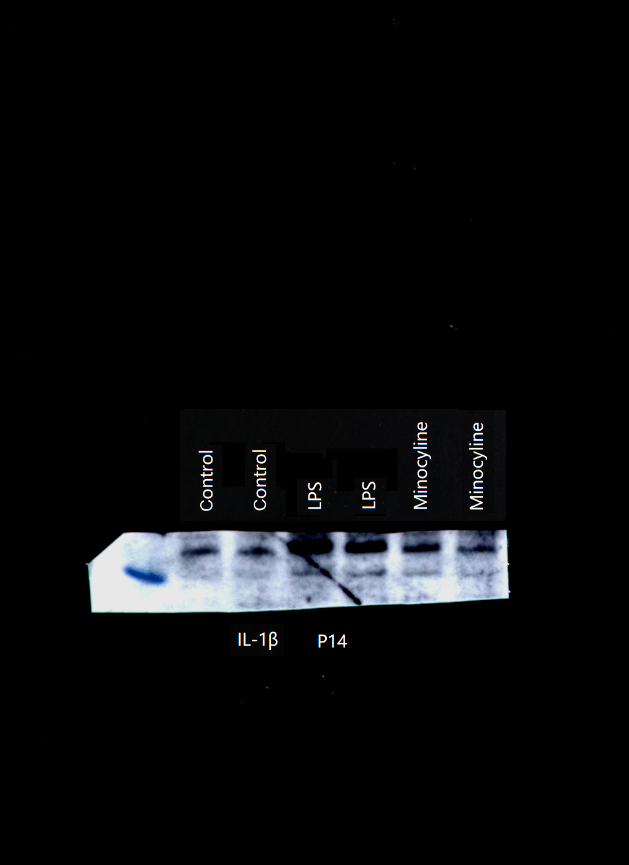

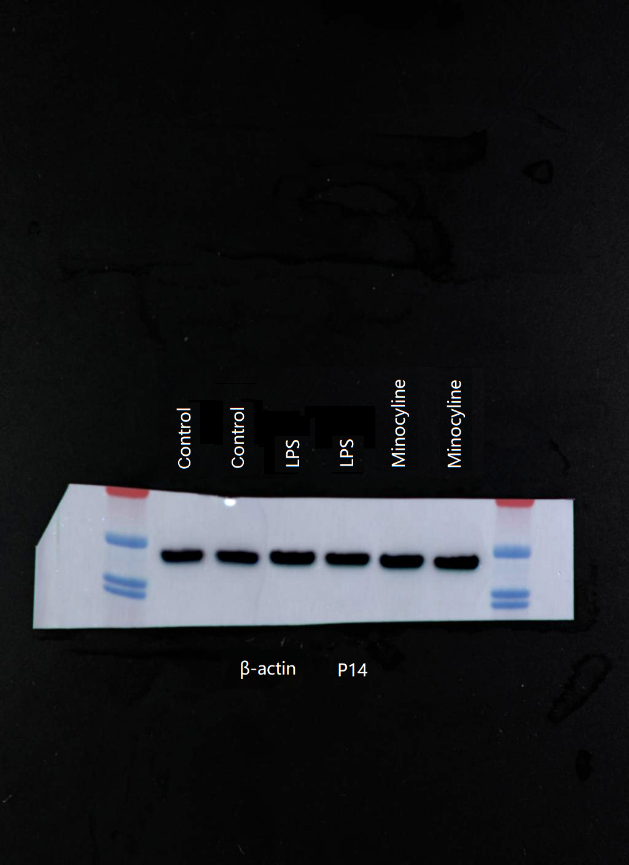


Original gels for Fig. S2 right panel.


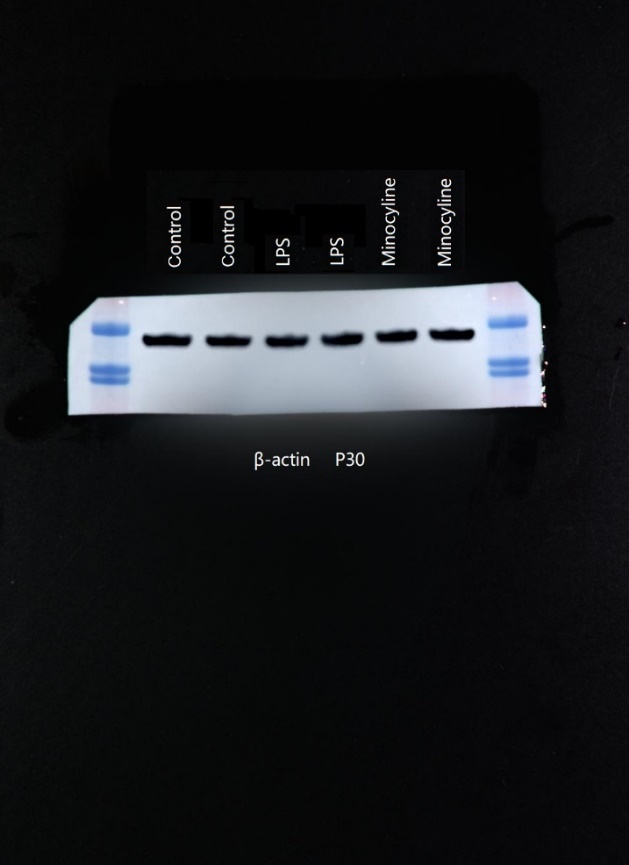

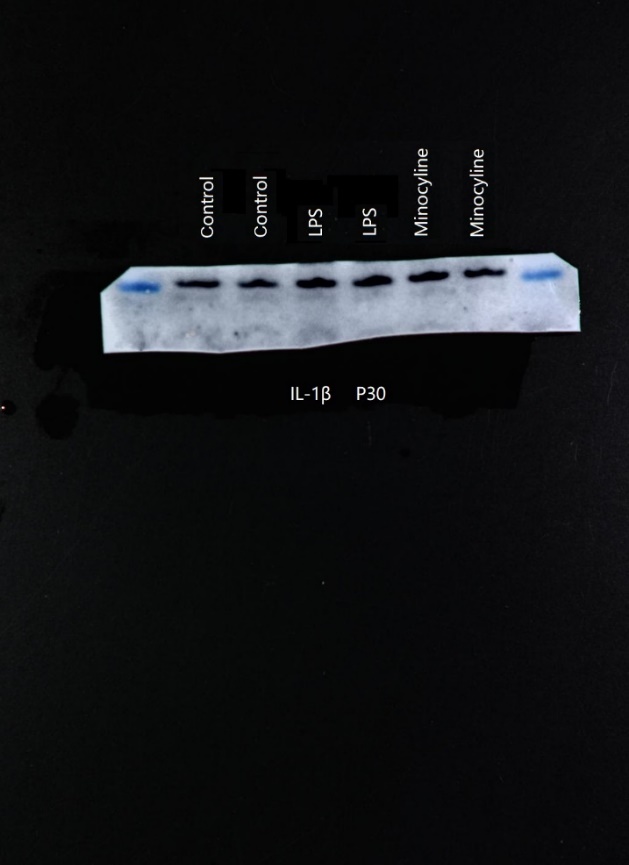


Original gels for Fig. S4B.


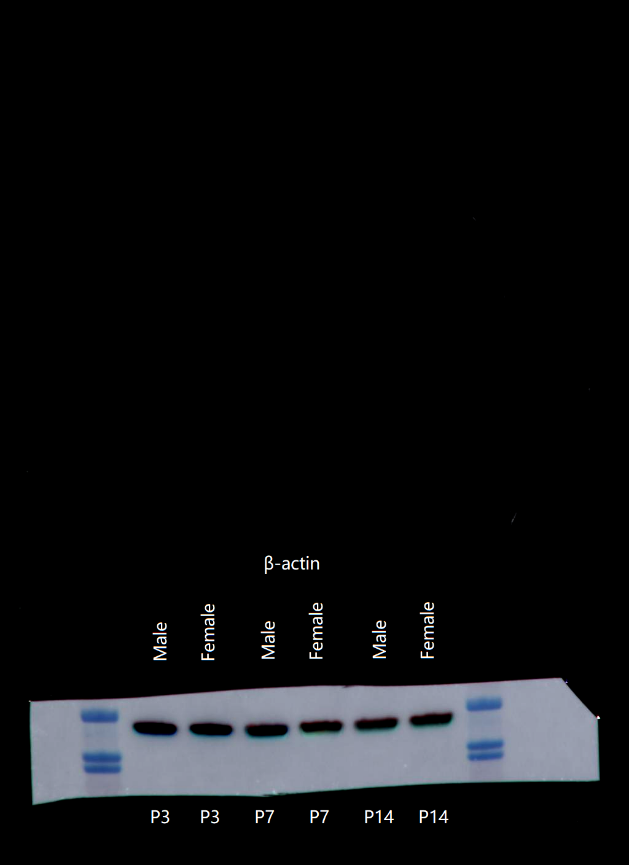

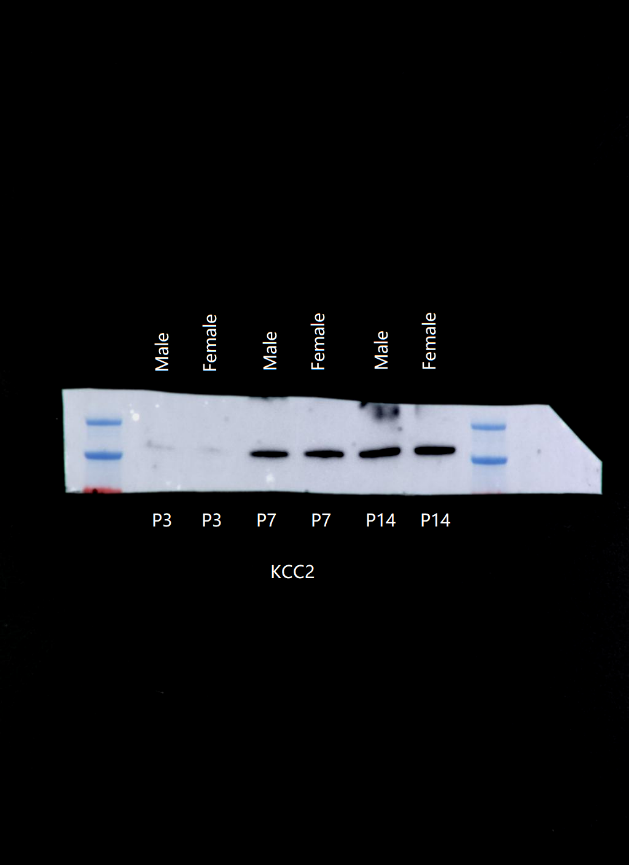


Original gels for Fig. S6 left panel.


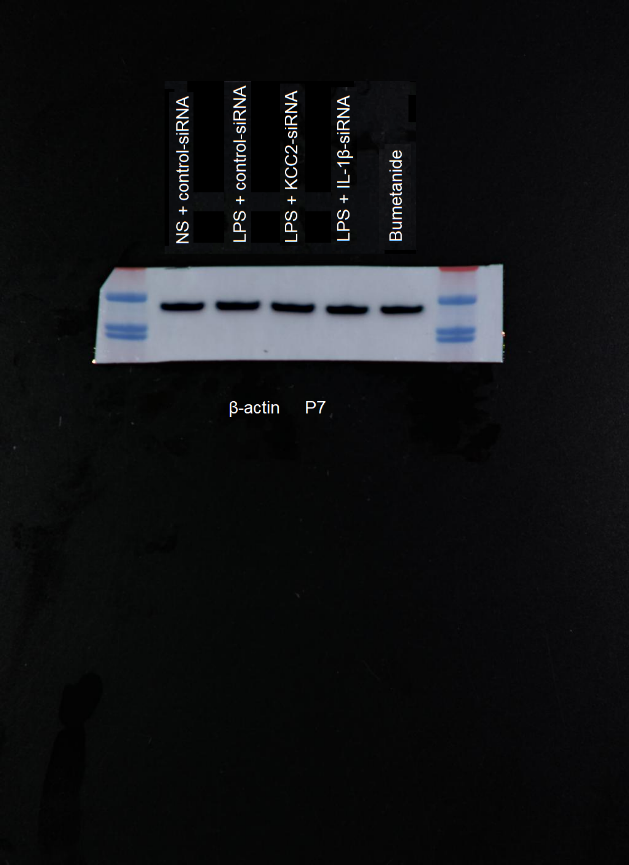

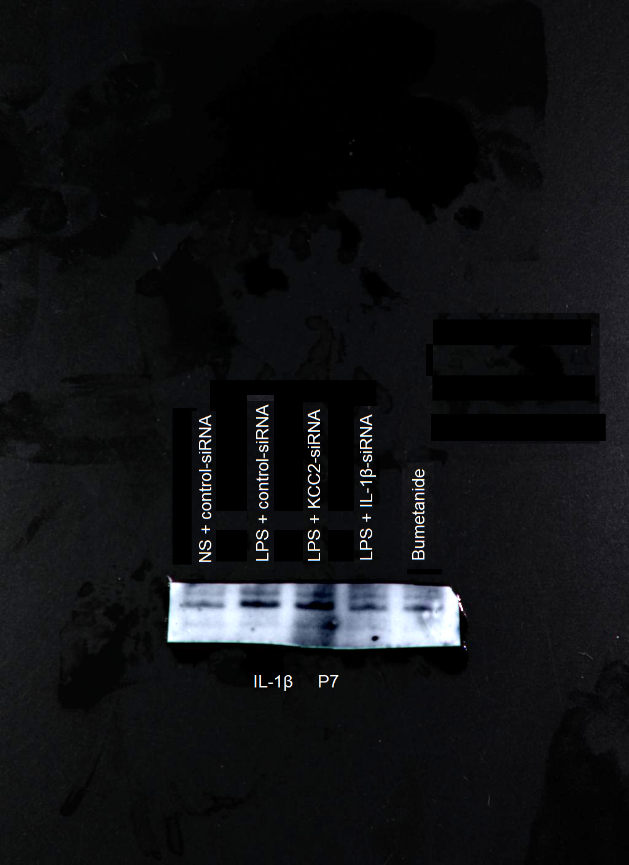


Original gels for Fig. S6 middle panel.


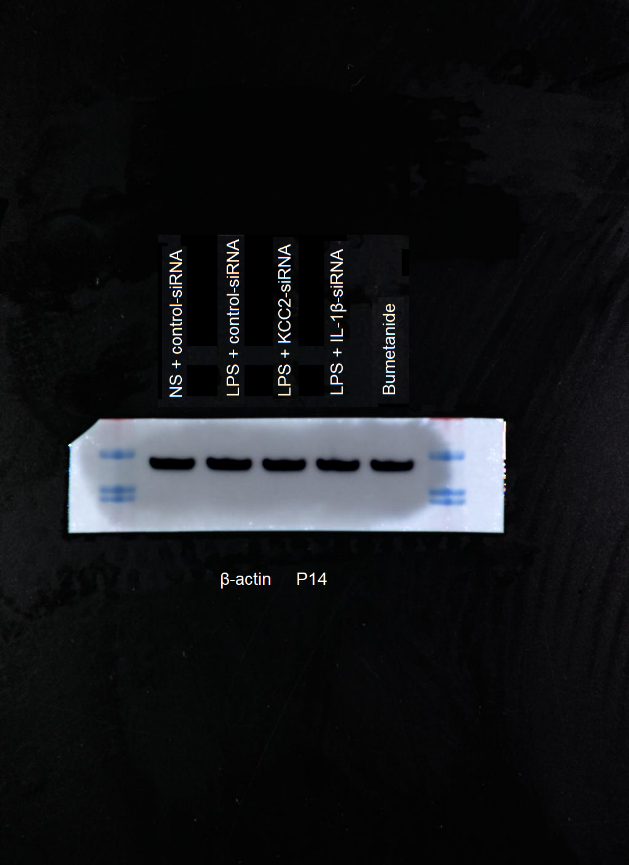

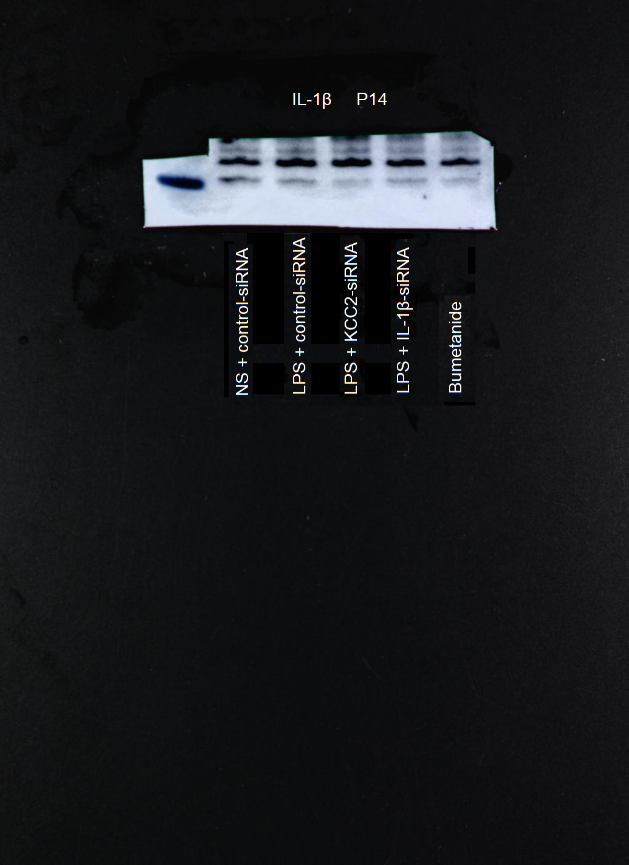


Original gels for Fig. S6 right panel.


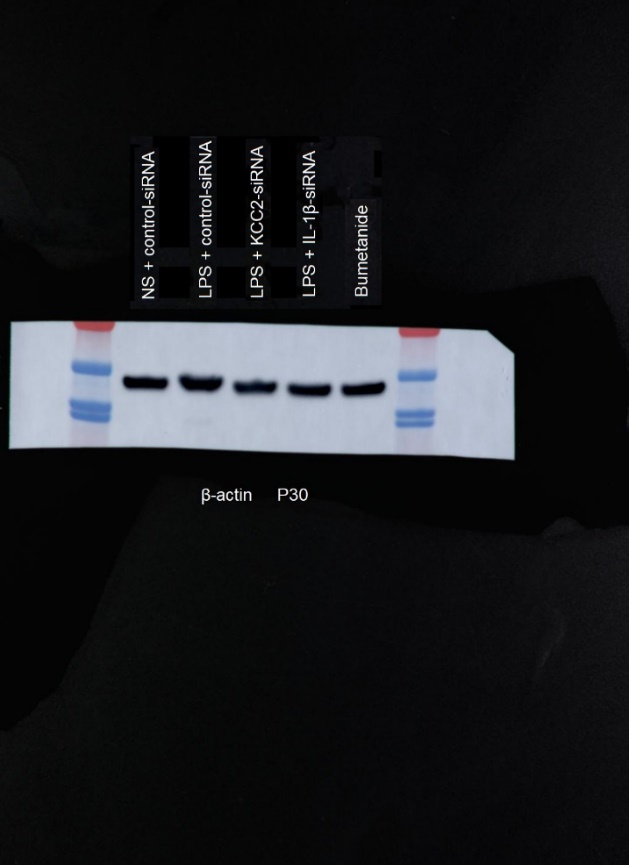

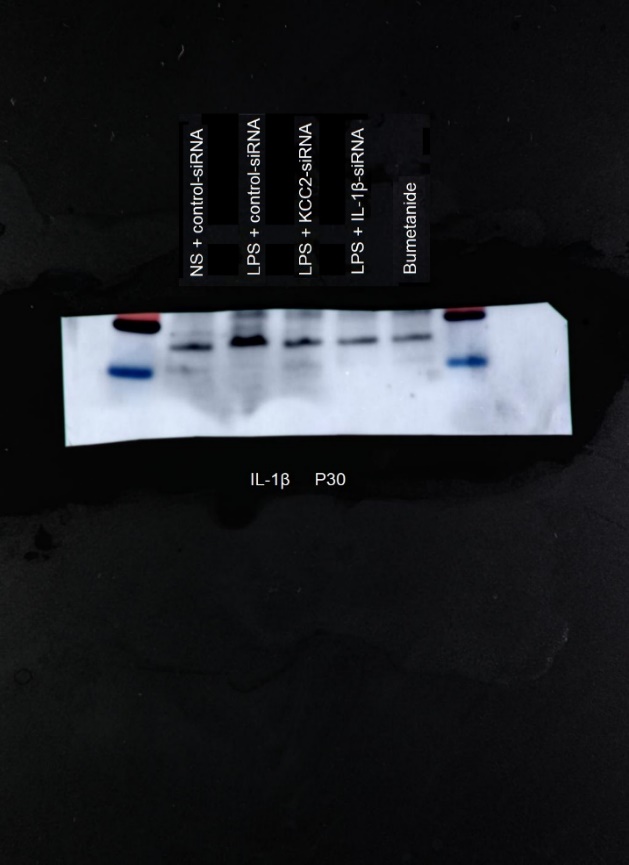


Original gels for Fig. S7 left panel.


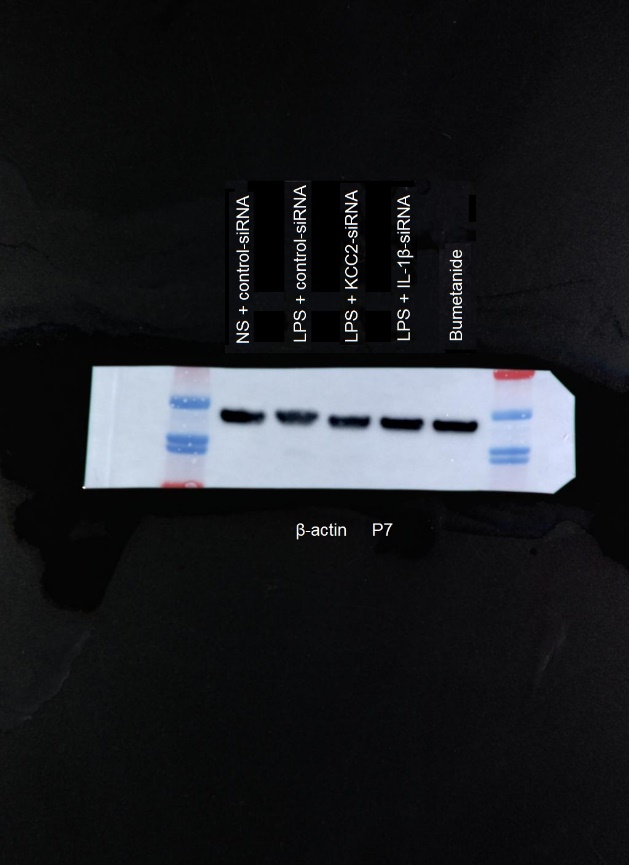

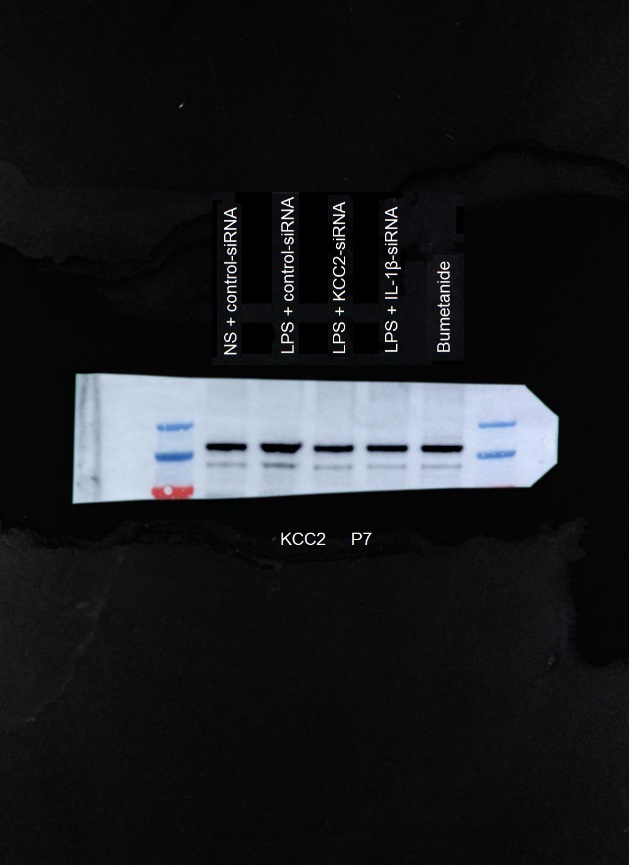


Original gels for Fig. S7 middle panel.


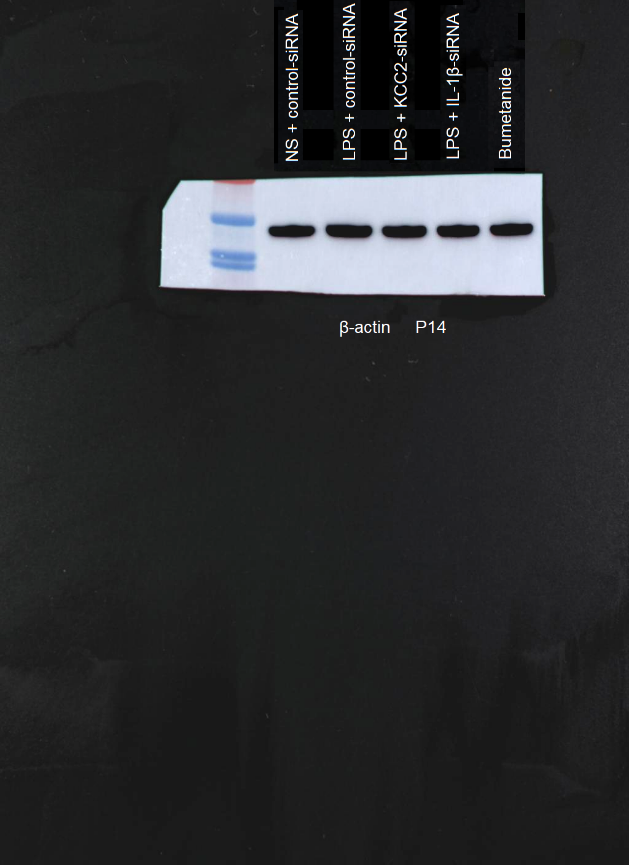

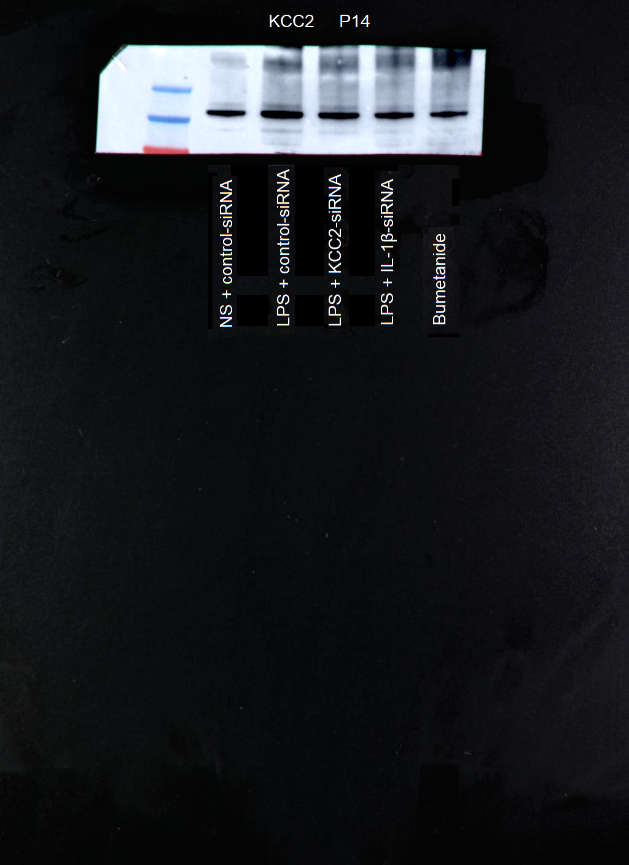


Original gels for Fig. S7 right panel.


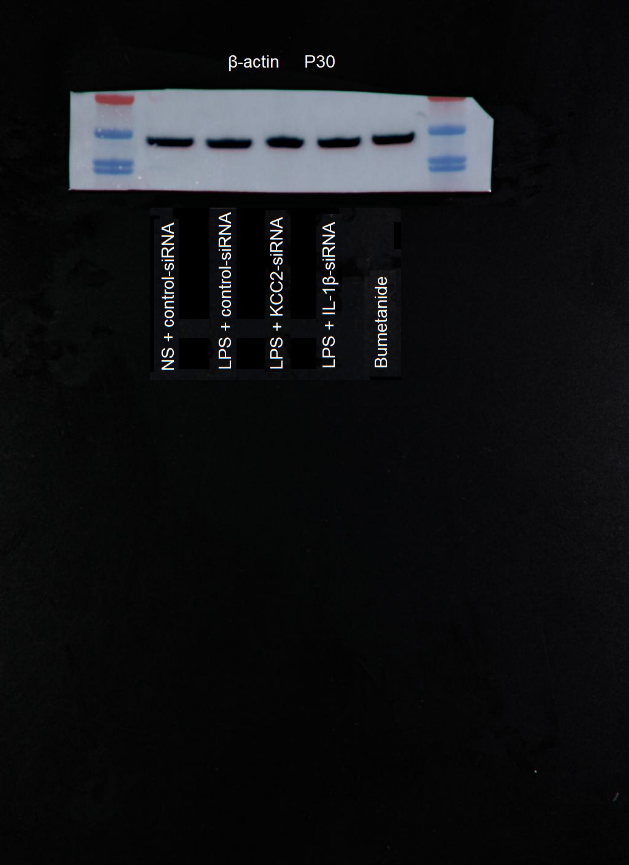

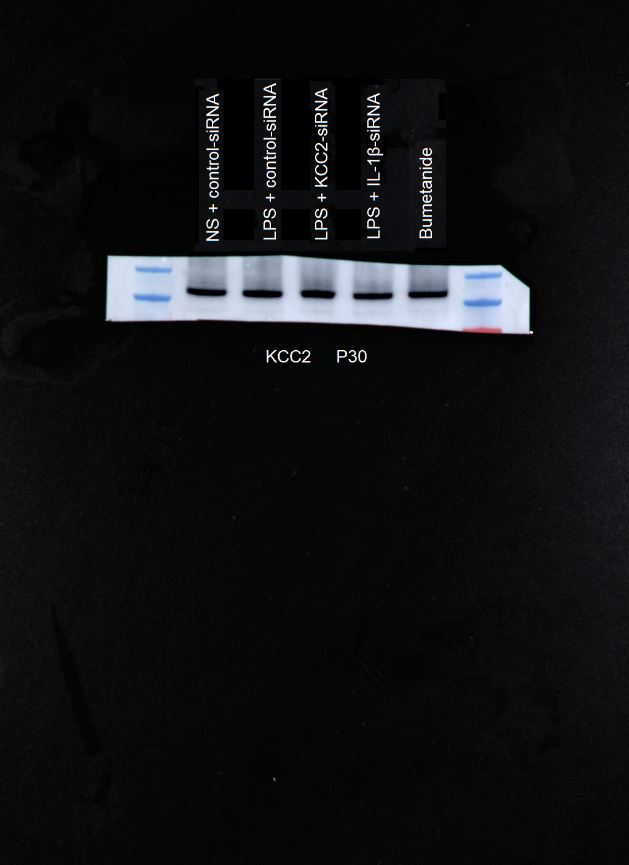

Supplement: Supplementary file 1 — Additional file 1. Original images for Western blotting results. [file 12916_2022_2434_MOESM1_ESM.docx]
