# Supplementary material for: Severe inflammation in new-borns induces long-term cognitive impairment by activation of IL-1β/KCC2 signaling during early development
Source: BMC Med. 2022 Jul 27;20:235. doi: 10.1186/s12916-022-02434-w (PMC9327322; doi:10.1186/s12916-022-02434-w)
Supplement: Supplementary file 2 — Additional file 2: Table S1-S11. Table. S1: Statistical information for Fig. 1. Table. S2: Statistical information for Fig. 2. Table. S3: Statistical information for Fig. 3. Table. S4: Statistical information for Fig. 4. Table. S5: Statistical information for Fig. 5. Table. S6: Statistical information for Supplementary figure 1. Table. S7: Statistical information for Supplementary figure 2. Table. S8: Statistical information for Supplementary figure 4. Table. S9: Statistical information for Supplementary figure 5. Table. S10: Statistical information for Supplementary figure 6. Table. S11: Statistical information for Supplementary figure 7. [file 12916_2022_2434_MOESM2_ESM.docx]

Supplementary table 1: Statistical information for Fig. 1.

| Panel | Group (n) | t, df | P | Test |
| --- | --- | --- | --- | --- |
| F | Control (15) vs. LPS (19) | 4.899, 32 | <0.0001 | Unpaired Student's t test |
| G | Control (15) vs. LPS (19) | 3.745, 32 | 0.0007 | Unpaired Student's t test |
| H | Control (15) vs. LPS (19) | 7.206, 32 | <0.0001 | Unpaired Student's t test |
| I | Control (15) vs. LPS (19) | 1.911, 32 | 0.0650 | Unpaired Student's t test |
| L | Control (15) vs. LPS (19) | 6.295, 32 | <0.0001 | Unpaired Student's t test |
| M | Control (15) vs. LPS (19) | 0.7244, 32 | 0.4741 | Unpaired Student's t test |
| B | ANOVA table | F (DFn, DFd) | P value |  |
|  | Interaction | F (6, 98) = 85.27 | P<0.0001 |  |
|  | Row Factor | F (6, 98) = 1482 | P<0.0001 |  |
|  | Column Factor | F (1, 98) = 401.6 | P<0.0001 |  |
|  | Bonferroni's multiple comparisons test | Mean Diff. | 95.00% CI of diff. | Adjusted P Value |
|  | 3 days | 0.2875 | -3.230 to 3.805 | >0.9999 |
|  | 5 days | 3.100 | -0.4170 to 6.617 | 0.1209 |
|  | 7 days | 4.662 | 1.155 to 8.170 | 0.0030 |
|  | 11 days | 4.975 | 1.458 to 8.492 | 0.0013 |
|  | 14 days | 4.363 | 0.8455 to 7.880 | 0.0066 |
|  | 21 days | 16.67 | 13.15 to 20.18 | <0.0001 |
|  | 30 days | 33.81 | 30.29 to 37.33 | <0.0001 |
| D | ANOVA table | F (DFn, DFd) | P value |  |
|  | Interaction | F (3, 64) = 0.4562 | P=0.7138 |  |
|  | Row Factor | F (3, 64) = 56.93 | P<0.0001 |  |
|  | Column Factor | F (1, 64) = 12.72 | P=0.0007 |  |
|  | Bonferroni's multiple comparisons test | Mean Diff. | 95.00% CI of diff. | Adjusted P Value |
|  | Day 1 | -6.230 | -17.55 to 5.086 | 0.6476 |
|  | Day 2 | -5.653 | -16.97 to 5.663 | 0.8152 |
|  | Day 3 | -7.337 | -18.65 to 3.978 | 0.4019 |
|  | Day 4 | -12.19 | -23.50 to -0.8716 | 0.0295 |
| K | ANOVA table | F (DFn, DFd) | P value |  |
|  | Interaction | F (4, 83) = 3.543 | P=0.0102 |  |
|  | Row Factor | F (4, 83) = 61.89 | P<0.0001 |  |
|  | Column Factor | F (1, 83) = 14.64 | P=0.0003 |  |
|  | Bonferroni's multiple comparisons test | Mean Diff. | 95.00% CI of diff. | Adjusted P Value |
|  | 120 | 0.5417 | -17.10 to 18.18 | >0.9999 |
|  | 142 | 1.636 | -16.00 to 19.28 | >0.9999 |
|  | 262 | 7.301 | -9.787 to 24.39 | >0.9999 |
|  | 284 | 16.54 | -0.5447 to 33.63 | 0.0627 |
|  | 404 | 30.17 | 13.08 to 47.25 | <0.0001 |

Supplementary table 2: Statistical information for Fig. 2.

| Panel | Group (n) | t, df | P | Test |
| --- | --- | --- | --- | --- |
| B-2h | Control (6) vs. LPS (6) |  | 0.0022 | Mann Whitney test |
| B-4h | Control (6) vs. LPS (6) |  | 0.0022 | Mann Whitney test |
| B-6h | Control (6) vs. LPS (6) |  | 0.0022 | Mann Whitney test |
| B-24h | Control (6) vs. LPS (6) | 0.6743,10 | 0.5154 | Unpaired Student's t test |
| C-6h | Control (6) vs. LPS (6) |  | 0.0022 | Mann Whitney test |
| C-P5 | Control (6) vs. LPS (6) | 0.4027,10 | 0.6957 | Unpaired Student's t test |
| C-P7 | Control (6) vs. LPS (6) | 0.6696,10 | 0.5183 | Unpaired Student's t test |
| C-P14 | Control (6) vs. LPS (6) | 0.5207,10 | 0.6139 | Unpaired Student's t test |
| D-2h | Control (6) vs. LPS (6) | 22.10,10 | <0.0001 | Unpaired Student's t test |
| D-4h | Control (6) vs. LPS (6) |  | 0.0022 | Mann Whitney test |
| D-6h | Control (6) vs. LPS (6) |  | 0.0022 | Mann Whitney test |
| D-24h | Control (6) vs. LPS (6) | 0.2875,10 | 0.7796 | Unpaired Student's t test |
| E-6h | Control (6) vs. LPS (6) |  | 0.0022 | Mann Whitney test |
| E-P5 | Control (6) vs. LPS (6) |  | 0.7771 | Mann Whitney test |
| E-P7 | Control (6) vs. LPS (6) | 0.7751,10 | 0.4562 | Unpaired Student's t test |
| E-P14 | Control (6) vs. LPS (6) | 0.1771,10 | 0.8630 | Unpaired Student's t test |
| F-2h | Control (6) vs. LPS (6) |  | 0.0022 | Mann Whitney test |
| F-4h | Control (6) vs. LPS (6) |  | 0.0022 | Mann Whitney test |
| F-6h | Control (6) vs. LPS (6) | 30.94,10 | <0.0001 | Unpaired Student's t test |
| F-24h | Control (6) vs. LPS (6) | 0.1130,10 | 0.9123 | Unpaired Student's t test |
| G-6h | Control (6) vs. LPS (6) |  | 0.0022 | Mann Whitney test |
| G-P5 | Control (6) vs. LPS (6) |  | 0.0022 | Mann Whitney test |
| G-P7 | Control (6) vs. LPS (6) |  | 0.0022 | Mann Whitney test |
| G-P14 | Control (6) vs. LPS (6) |  | 0.0022 | Mann Whitney test |
| G-P30 | Control (6) vs. LPS (6) |  | 0.0022 | Mann Whitney test |

Supplementary table 3: Statistical information for Fig. 3.

| Panel | Group (n) | t, df | P | Test |
| --- | --- | --- | --- | --- |
| B | Control (6) vs. LPS (6) | 6.741, 10 | <0.0001 | Unpaired Student's t test |
| C | ANOVA table | F (DFn, DFd) | P value |  |
|  | Interaction | F (6, 216) = 1.486 | P=0.1841 |  |
|  | Row Factor | F (3, 216) = 48.03 | P<0.0001 |  |
|  | Column Factor | F (2, 216) = 19.49 | P<0.0001 |  |
|  | Bonferroni's multiple comparisons test | Mean Diff. | 95.00% CI of diff. | Adjusted P Value |
| C-Day 1 | NS + Control-siRNA vs. LPS + Control-siRNA | -10.25 | -21.16 to 0.6561 | 0.0730 |
|  | NS + Control-siRNA vs. LPS + IL-1β-siRNA | -6.767 | -17.46 to 3.924 | 0.3846 |
|  | LPS + Control-siRNA vs. LPS + IL-1β-siRNA | 3.483 | -6.382 to 13.35 | >0.9999 |
| C-Day 2 | NS + Control-siRNA vs. LPS + Control-siRNA | -8.006 | -18.91 to 2.900 | 0.2338 |
|  | NS + Control-siRNA vs. LPS + IL-1β-siRNA | -1.460 | -12.15 to 9.231 | >0.9999 |
|  | LPS + Control-siRNA vs. LPS + IL-1β-siRNA | 6.546 | -3.319 to 16.41 | 0.3325 |
| C-Day 3 | NS + Control-siRNA vs. LPS + Control-siRNA | -12.93 | -23.84 to -2.024 | 0.0139 |
|  | NS + Control-siRNA vs. LPS + IL-1β-siRNA | -6.206 | -16.90 to 4.485 | 0.4884 |
|  | LPS + Control-siRNA vs. LPS + IL-1β-siRNA | 6.724 | -3.141 to 16.59 | 0.3046 |
| C-Day 4 | NS + Control-siRNA vs. LPS + Control-siRNA | -24.77 | -35.68 to -13.87 | <0.0001 |
|  | NS + Control-siRNA vs. LPS + IL-1β-siRNA | -11.66 | -22.35 to -0.9706 | 0.0273 |
|  | LPS + Control-siRNA vs. LPS + IL-1β-siRNA | 13.11 | 3.246 to 22.98 | 0.0046 |
| D | ANOVA summary |  |  |  |
|  | P value | <0.0001 |  |  |
|  | Treatment (between columns) | F (2, 56) = 60.32 |  |  |
|  | Tukey's multiple comparisons test | Mean Diff. | 95.00% CI of diff. | Adjusted P Value |
|  | NS + Control-siRNA vs. LPS + Control-siRNA | 14.96 | 11.67 to 18.24 | <0.0001 |
|  | NS + Control-siRNA vs. LPS + IL-1β-siRNA | 9.253 | 6.088 to 12.42 | <0.0001 |
|  | LPS + Control-siRNA vs. LPS + IL-1β-siRNA | -5.704 | -8.616 to -2.793 | <0.0001 |
| E | P value | <0.0001 |  |  |
|  | Treatment (between columns) | F (2, 56) = 31.80 |  |  |
|  | Tukey's multiple comparisons test | Mean Diff. | 95.00% CI of diff. | Adjusted P Value |
|  | NS + Control-siRNA vs. LPS + Control-siRNA | 11.26 | 7.834 to 14.68 | <0.0001 |
|  | NS + Control-siRNA vs. LPS + IL-1β-siRNA | 7.500 | 4.201 to 10.80 | <0.0001 |
|  | LPS + Control-siRNA vs. LPS+IL-1β-siRNA | -3.758 | -6.792 to -0.7228 | 0.0116 |
| F | P value | <0.0001 |  |  |
|  | Treatment (between columns) | F (2, 58) = 23.49 |  |  |
|  | Tukey's multiple comparisons test | Mean Diff. | 95.00% CI of diff. | Adjusted P Value |
|  | NS + Control-siRNA vs. LPS + Control-siRNA | 2.571 | 1.668 to 3.473 | <0.0001 |
|  | NS + Control-siRNA vs. LPS + IL-1β-siRNA | 1.429 | 0.5619 to 2.296 | 0.0006 |
|  | LPS + Control-siRNA vs. LPS + IL-1β-siRNA | -1.142 | -1.970 to -0.3136 | 0.0044 |
| G | P value | 0.6105 |  |  |
|  | Treatment (between columns) | F (2, 51) = 0.4984 |  |  |
|  | Tukey's multiple comparisons test | Mean Diff. | 95.00% CI of diff. | Adjusted P Value |
|  | NS + Control-siRNA vs. LPS + Control-siRNA | 4.400 | -8.044 to 16.84 | 0.6718 |
|  | NS + Control-siRNA vs. LPS + IL-1β-siRNA | 0.8565 | -11.10 to 12.82 | 0.9837 |
|  | LPS + Control-siRNA vs. LPS + IL-1β-siRNA | -3.543 | -13.63 to 6.543 | 0.6751 |
| H | ANOVA table | F (DFn, DFd) | P value |  |
|  | Interaction | F (8, 280) = 3.758 | P=0.0003 |  |
|  | Row Factor | F (4, 280) = 291.0 | P<0.0001 |  |
|  | Column Factor | F (2, 280) = 12.07 | P<0.0001 |  |
|  | Bonferroni's multiple comparisons test | Mean Diff. | 95.00% CI of diff. | Adjusted P Value |
| H-120 | NS + Control-siRNA vs. LPS + Control-siRNA | 0.04550 | -9.753 to 9.844 | >0.9999 |
|  | NS + Control-siRNA vs. LPS + IL-1β-siRNA | -0.07879 | -9.521 to 9.363 | >0.9999 |
|  | LPS + Control-siRNA vs. LPS + IL-1β-siRNA | -0.1243 | -8.810 to 8.561 | >0.9999 |
| H-142 | NS + Control-siRNA vs. LPS + Control-siRNA | 0.009171 | -9.789 to 9.808 | >0.9999 |
|  | NS + Control-siRNA vs. LPS + IL-1β-siRNA | -0.1013 | -9.543 to 9.341 | >0.9999 |
|  | LPS + Control-siRNA vs. LPS + IL-1β-siRNA | -0.1105 | -8.796 to 8.575 | >0.9999 |
| H-262 | NS + Control-siRNA vs. LPS + Control-siRNA | 6.312 | -3.487 to 16.11 | 0.3658 |
|  | NS + Control-siRNA vs. LPS + IL-1β-siRNA | 2.925 | -6.517 to 12.37 | >0.9999 |
|  | LPS + Control-siRNA vs. LPS + IL-1β-siRNA | -3.387 | -12.07 to 5.299 | >0.9999 |
| H-284 | NS + Control-siRNA vs. LPS + Control-siRNA | 11.34 | 1.542 to 21.14 | 0.0170 |
|  | NS + Control-siRNA vs. LPS + IL-1β-siRNA | 6.335 | -3.107 to 15.78 | 0.3218 |
|  | LPS + Control-siRNA vs. LPS + IL-1β-siRNA | -5.005 | -13.69 to 3.680 | 0.4987 |
| H-404 | NS + Control-siRNA vs. LPS + Control-siRNA | 26.60 | 16.80 to 36.40 | <0.0001 |
|  | NS + Control-siRNA vs. LPS + IL-1β-siRNA | 11.73 | 2.291 to 21.18 | 0.0090 |
|  | LPS + Control-siRNA vs. LPS+IL-1β-siRNA | -14.87 | -23.55 to -6.184 | 0.0001 |
| I | P value | <0.0001 |  |  |
|  | Treatment (between columns) | F (2, 56) = 13.54 |  |  |
|  | Tukey's multiple comparisons test | Mean Diff. | 95.00% CI of diff. | Adjusted P Value |
|  | NS + Control-siRNA vs. LPS + Control-siRNA | 29.44 | 15.14 to 43.75 | <0.0001 |
|  | NS + Control-siRNA vs. LPS + IL-1β-siRNA | 9.515 | -4.267 to 23.30 | 0.2287 |
|  | LPS + Control-siRNA vs. LPS + IL-1β-siRNA | -19.93 | -32.61 to -7.250 | 0.0011 |
| J | P value | 0.7863 |  |  |
|  | Treatment (between columns) | F (2, 56) = 0.2415 |  |  |
|  | Tukey's multiple comparisons test | Mean Diff. | 95.00% CI of diff. | Adjusted P Value |
|  | NS + Control-siRNA vs. LPS + Control-siRNA | -0.2713 | -13.20 to 12.66 | 0.9986 |
|  | NS + Control-siRNA vs. LPS + IL-1β-siRNA | 2.733 | -9.725 to 15.19 | 0.8579 |
|  | LPS + Control-siRNA vs. LPS + IL-1β-siRNA | 3.004 | -8.456 to 14.46 | 0.8037 |

Supplementary table 4: Statistical information for Fig. 4.

| Panel | Group (n) | t, df | P | Test |
| --- | --- | --- | --- | --- |
| B-P7 | Control (6) vs. LPS (6) | 7.371, 10 | <0.0001 | Unpaired Student's t test |
| B-P14 | Control (6) vs. LPS (6) | 5.025, 10 | 0.0005 | Unpaired Student's t test |
| B-P30 | Control (6) vs. LPS (6) | 1.278, 10 | 0.2301 | Unpaired Student's t test |
| D | Control (6) vs. LPS (6) | 5.980, 10 | 0.0001 | Unpaired Student's t test |
| F | P value | 0.0091 |  |  |
|  | Treatment (between columns) | F (3, 50) = 4.285 |  |  |
|  | Tukey's multiple comparisons test | Mean Diff. | 95.00% CI of diff. | Adjusted P Value |
|  | NS + Control-siRNA vs. LPS + Control-siRNA | 8.654 | -0.3811 to 17.69 | 0.0650 |
|  | NS + Control-siRNA vs. LPS + IL-1β-siRNA | -2.656 | -10.64 to 5.330 | 0.8132 |
|  | NS + Control-siRNA vs. LPS + KCC2-siRNA | -1.167 | -9.415 to 7.081 | 0.9817 |
|  | LPS + Control-siRNA vs. LPS + IL-1β-siRNA | -11.31 | -20.11 to -2.514 | 0.0067 |
|  | LPS + Control-siRNA vs. LPS + KCC2-siRNA | -9.821 | -18.86 to -0.7861 | 0.0282 |
|  | LPS + IL-1β-siRNA vs. LPS + KCC2-siRNA | 1.489 | -6.497 to 9.475 | 0.9597 |
| G | P value | 0.0228 |  |  |
|  | Treatment (between columns) | F (3, 50) = 3.472 |  |  |
|  | Tukey's multiple comparisons test | Mean Diff. | 95.00% CI of diff. | Adjusted P Value |
|  | NS + Control-siRNA vs. LPS + Control-siRNA | 8.317 | 0.3700 to 16.26 | 0.0370 |
|  | NS + Control-siRNA vs. LPS + IL-1β-siRNA | 0.2274 | -6.906 to 7.360 | 0.9998 |
|  | NS + Control-siRNA vs. LPS + KCC2-siRNA | 0.2274 | -6.906 to 7.360 | 0.9998 |
|  | LPS + Control-siRNA vs. LPS + IL-1β-siRNA | -8.090 | -15.93 to -0.2537 | 0.0406 |
|  | LPS + Control-siRNA vs. LPS + KCC2-siRNA | -8.090 | -15.93 to -0.2537 | 0.0406 |
|  | LPS + IL-1β-siRNA vs. LPS + KCC2-siRNA | 0.000 | -7.009 to 7.009 | >0.9999 |
| H | P value | 0.0001 |  |  |
|  | Treatment (between columns) | F (3, 50) = 8.340 |  |  |
|  | Tukey's multiple comparisons test | Mean Diff. | 95.00% CI of diff. | Adjusted P Value |
|  | NS + Control-siRNA vs. LPS + Control-siRNA | 3.543 | 1.562 to 5.523 | <0.0001 |
|  | NS + Control-siRNA vs. LPS + IL-1β-siRNA | 0.7095 | -1.068 to 2.487 | 0.7147 |
|  | NS + Control-siRNA vs. LPS + KCC2-siRNA | 0.7095 | -1.068 to 2.487 | 0.7147 |
|  | LPS + Control-siRNA vs. LPS + IL-1β-siRNA | -2.833 | -4.786 to -0.8805 | 0.0018 |
|  | LPS + Control-siRNA vs. LPS+KCC2-siRNA | -2.833 | -4.786 to -0.8805 | 0.0018 |
|  | LPS+IL-1β-siRNA vs. LPS+KCC2-siRNA | 0.000 | -1.747 to 1.747 | >0.9999 |
| I | P value | 0.0058 |  |  |
|  | Treatment (between columns) | F (3, 50) = 4.694 |  |  |
|  | Tukey's multiple comparisons test | Mean Diff. | 95.00% CI of diff. | Adjusted P Value |
|  | NS + Control-siRNA vs. LPS + Control-siRNA | -7.464 | -13.43 to -1.498 | 0.0087 |
|  | NS + Control-siRNA vs. LPS + IL-1β-siRNA | -3.786 | -9.140 to 1.568 | 0.2499 |
|  | NS + Control-siRNA vs. LPS + KCC2-siRNA | -6.166 | -11.52 to -0.8123 | 0.0180 |
|  | LPS + Control-siRNA vs. LPS + IL-1β-siRNA | 3.677 | -2.204 to 9.559 | 0.3545 |
|  | LPS + Control-siRNA vs. LPS + KCC2-siRNA | 1.297 | -4.584 to 7.179 | 0.9358 |
|  | LPS + IL-1β-siRNA vs. LPS + KCC2-siRNA | -2.380 | -7.641 to 2.881 | 0.6283 |
| K | P value | 0.0004 |  |  |
|  | Treatment (between columns) | F (3, 50) = 7.259 |  |  |
|  | Tukey's multiple comparisons test | Mean Diff. | 95.00% CI of diff. | Adjusted P Value |
|  | NS + Control-siRNA vs. LPS + Control-siRNA | 35.36 | 13.19 to 57.54 | 0.0005 |
|  | NS + Control-siRNA vs. LPS + IL-1β-siRNA | 4.658 | -15.25 to 24.56 | 0.9246 |
|  | NS + Control-siRNA vs. LPS+KCC2-siRNA | 3.338 | -16.57 to 23.24 | 0.9702 |
|  | LPS + Control-siRNA vs. LPS + IL-1β-siRNA | -30.71 | -52.57 to -8.839 | 0.0027 |
|  | LPS + Control-siRNA vs. LPS + KCC2-siRNA | -32.03 | -53.89 to -10.16 | 0.0016 |
|  | LPS + IL-1β-siRNA vs. LPS + KCC2-siRNA | -1.320 | -20.88 to 18.24 | 0.9979 |
| L | P value | 0.4111 |  |  |
|  | Treatment (between columns) | F (3, 50) = 0.9770 |  |  |
|  | Tukey's multiple comparisons test | Mean Diff. | 95.00% CI of diff. | Adjusted P Value |
|  | NS + Control-siRNA vs. LPS + Control-siRNA | -1.945 | -18.19 to 14.30 | 0.9887 |
|  | NS + Control-siRNA vs. LPS + IL-1β-siRNA | -6.068 | -20.65 to 8.514 | 0.6876 |
|  | NS + Control-siRNA vs. LPS + KCC2-siRNA | -8.611 | -23.19 to 5.970 | 0.4050 |
|  | LPS + Control-siRNA vs. LPS+IL-1β-siRNA | -4.123 | -20.14 to 11.90 | 0.9027 |
|  | LPS + Control-siRNA vs. LPS + KCC2-siRNA | -6.667 | -22.69 to 9.353 | 0.6876 |
|  | LPS + IL-1β-siRNA vs. LPS + KCC2-siRNA | -2.544 | -16.87 to 11.78 | 0.9649 |

Supplementary table 5: Statistical information for Fig. 5.

| Panel | Group (n) | t, df | P | Test |
| --- | --- | --- | --- | --- |
| D | Control (12) vs. LPS (10) | 4.154, 20 | 0.0005 | Unpaired Student's t test |
| E | Control (14) vs. LPS (9) | 2.926, 21 | 0.0081 | Unpaired Student's t test |
| H | Control (7) vs. LPS (7) | 3.766, 12 | 0.0027 | Unpaired Student's t test |
| I | Control (13) vs. LPS (10) | 2.914, 21 | 0.0083 | Unpaired Student's t test |
| K | Control (6) vs. LPS (7) | 0.7933, 11 | 0.4444 | Unpaired Student's t test |
| L | Control (8) vs. LPS (10) | 0.2086, 16 | 0.8374 | Unpaired Student's t test |
| O | P value | 0.0121 |  |  |
|  | Treatment (between columns) | F (2, 19) = 5.614 |  |  |
|  | Tukey's multiple comparisons test | Mean Diff. | 95.00% CI of diff. | Adjusted P Value |
|  | LPS + Control-siRNA vs. LPS + IL-1β-siRNA | -14.15 | -26.22 to -2.082 | 0.0201 |
|  | LPS + Control-siRNA vs. LPS + KCC2-siRNA | -13.05 | -25.12 to -0.9809 | 0.0328 |
|  | LPS + IL-1β-siRNA vs. LPS + KCC2-siRNA | 1.101 | -11.36 to 13.56 | 0.9726 |
| P | P value | 0.0126 |  |  |
|  | Treatment (between columns) | F (2, 23) = 5.324 |  |  |
|  | Tukey's multiple comparisons test | Mean Diff. | 95.00% CI of diff. | Adjusted P Value |
|  | LPS + Control-siRNA vs. LPS + IL-1β-siRNA | -4.919 | -9.287 to -0.5503 | 0.0253 |
|  | LPS + Control-siRNA vs. LPS + KCC2-siRNA | -5.574 | -10.44 to -0.7080 | 0.0227 |
|  | LPS+IL-1β-siRNA vs. LPS+KCC2-siRNA | -0.6549 | -5.200 to 3.890 | 0.9310 |
| R | P value | 0.0045 |  |  |
|  | Treatment (between columns) | F (2, 15) = 7.921 |  |  |
|  | Tukey's multiple comparisons test | Mean Diff. | 95.00% CI of diff. | Adjusted P Value |
|  | LPS + Control-siRNA vs. LPS + IL-1β-siRNA | -11.02 | -20.35 to -1.689 | 0.0201 |
|  | LPS + Control-siRNA vs. LPS + KCC2-siRNA | -13.70 | -23.52 to -3.883 | 0.0066 |
|  | LPS + IL-1β-siRNA vs. LPS + KCC2-siRNA | -2.684 | -12.84 to 7.472 | 0.7749 |
| S | P value | 0.0065 |  |  |
|  | Treatment (between columns) | F (2, 21) = 6.457 |  |  |
|  | Tukey's multiple comparisons test | Mean Diff. | 95.00% CI of diff. | Adjusted P Value |
|  | LPS + Control-siRNA vs. LPS + IL-1β-siRNA | -6.992 | -13.07 to -0.9121 | 0.0224 |
|  | LPS + Control-siRNA vs. LPS + KCC2-siRNA | -8.025 | -14.33 to -1.720 | 0.0113 |
|  | LPS + IL-1β-siRNA vs. LPS + KCC2-siRNA | -1.033 | -7.509 to 5.442 | 0.9150 |

Supplementary table 6: Statistical information for Supplementary figure 1.

| Panel | Group (n) | t, df | P | Test |
| --- | --- | --- | --- | --- |
| A-male | Control (9) vs. LPS (9) | 3.461, 16 | 0.0032 | Unpaired Student's t test |
| A-female | Control (6) vs. LPS (10) | 3.200, 14 | 0.0064 | Unpaired Student's t test |
| B-male | Control (9) vs. LPS (9) | 3.241, 16 | 0.0051 | Unpaired Student's t test |
| B-female | Control (6) vs. LPS (10) | 2.192, 14 | 0.0458 | Unpaired Student's t test |
| C-male | Control (9) vs. LPS (9) | 5.091, 16 | 0.0001 | Unpaired Student's t test |
| C-female | Control (6) vs. LPS (10) | 4.791, 14 | 0.0003 | Unpaired Student's t test |
| D-male | Control (9) vs. LPS (9) | 4.238, 16 | 0.0006 | Unpaired Student's t test |
| D-female | Control (6) vs. LPS (10) | 4.292, 14 | 0.0007 | Unpaired Student's t test |
| E-male | Control (9) vs. LPS (9) | 1.069, 16 | 0.3010 | Unpaired Student's t test |
| E-female | Control (6) vs. LPS (10) | 0.06564, 14 | 0.9486 | Unpaired Student's t test |

Supplementary table 7: Statistical information for Supplementary figure 2.

| Panel | Group (n) | t, df | P | Test |
| --- | --- | --- | --- | --- |
| P7 | Control (6) vs. LPS (6) | 10.15, 10 | <0.0001 | Unpaired Student's t test |
| P14 | Control (6) vs. LPS (6) | 8.292, 10 | <0.0001 | Unpaired Student's t test |
| P30 | Control (6) vs. LPS (6) | 4.500, 10 | 0.0011 | Unpaired Student's t test |

Supplementary table 8. Statistical information for Supplementary figure 4.

| Panel | Group (n) | t, df | P | Test |
| --- | --- | --- | --- | --- |
| A-P3 | Control (6) vs. LPS (6) | 0.5485, 10 | 0.5954 | Unpaired Student's t test |
| A-P7 | Control (6) vs. LPS (6) | 1.776, 10 | 0.1061 | Unpaired Student's t test |
| A-P14 | Control (6) vs. LPS (6) | 0.4606, 10 | 0.6550 | Unpaired Student's t test |
| B-P3 | Control (4) vs. LPS (4) | 0.3925, 6 | 0.7083 | Unpaired Student's t test |
| B-P7 | Control (4) vs. LPS (4) | 0.09675, 6 | 0.9261 | Unpaired Student's t test |
| B-P14 | Control (4) vs. LPS (4) | 0.9565, 6 | 0.3758 | Unpaired Student's t test |

Supplementary table 9: Statistical information for Supplementary figure 5.

| A-P7 | P value | <0.0001 |  |  |
| --- | --- | --- | --- | --- |
|  | Treatment (between columns) | F (3, 24) = 32.23 |  |  |
|  | Tukey's multiple comparisons test | Mean Diff. | 95.00% CI of diff. | Adjusted P Value |
|  | NS + Control-siRNA vs. LPS + Control-siRNA | -8.266 | -10.80 to -5.734 | <0.0001 |
|  | NS + Control-siRNA vs. LPS + IL-1β-siRNA | -2.999 | -5.354 to -0.6438 | 0.0090 |
|  | NS + Control-siRNA vs. LPS + KCC2-siRNA | -6.232 | -8.664 to -3.799 | <0.0001 |
|  | LPS + Control-siRNA vs. LPS + IL-1β-siRNA | 5.267 | 2.810 to 7.725 | <0.0001 |
|  | LPS + Control-siRNA vs. LPS + KCC2-siRNA | 2.035 | -0.4972 to 4.566 | 0.1473 |
|  | LPS + IL-1β-siRNA vs. LPS + KCC2-siRNA | -3.233 | -5.588 to -0.8776 | 0.0047 |
| A-P14 | P value | <0.0001 |  |  |
|  | Treatment (between columns) | F (3, 26) = 17.12 |  |  |
|  | Tukey's multiple comparisons test | Mean Diff. | 95.00% CI of diff. | Adjusted P Value |
|  | NS + Control-siRNA vs. LPS + Control-siRNA | -7.087 | -9.929 to -4.245 | <0.0001 |
|  | NS + Control-siRNA vs. LPS + IL-1β-siRNA | -2.992 | -5.834 to -0.1497 | 0.0364 |
|  | NS + Control-siRNA vs. LPS + KCC2-siRNA | -5.296 | -8.365 to -2.226 | 0.0004 |
|  | LPS + Control-siRNA vs. LPS + IL-1β-siRNA | 4.095 | 1.253 to 6.937 | 0.0028 |
|  | LPS + Control-siRNA vs. LPS + KCC2-siRNA | 1.792 | -1.278 to 4.861 | 0.3955 |
|  | LPS + IL-1β-siRNA vs. LPS + KCC2-siRNA | -2.304 | -5.374 to 0.7659 | 0.1932 |
| A-P30 | P value | <0.0001 |  |  |
|  | Treatment (between columns) | F (3, 20) = 16.70 |  |  |
|  | Tukey's multiple comparisons test | Mean Diff. | 95.00% CI of diff. | Adjusted P Value |
|  | NS + Control-siRNA vs. LPS + Control-siRNA | NS + Control-siRNA vs. LPS + Control-siRNA | NS + Control-siRNA vs. LPS + Control-siRNA | <0.0001 |
|  | NS + Control-siRNA vs. LPS + IL-1β-siRNA | NS + Control-siRNA vs. LPS + IL-1β-siRNA | NS + Control-siRNA vs. LPS + IL-1β-siRNA | 0.0079 |
|  | NS + Control-siRNA vs. LPS + KCC2-siRNA | NS + Control-siRNA vs. LPS + KCC2-siRNA | NS + Control-siRNA vs. LPS + KCC2-siRNA | 0.0001 |
|  | LPS + Control-siRNA vs. LPS + IL-1β-siRNA | LPS + Control-siRNA vs. LPS + IL-1β-siRNA | LPS + Control-siRNA vs. LPS + IL-1β-siRNA | 0.0367 |
|  | LPS + Control-siRNA vs. LPS+KCC2-siRNA | LPS + Control-siRNA vs. LPS + KCC2-siRNA | LPS + Control-siRNA vs. LPS + KCC2-siRNA | 0.6796 |
|  | LPS + IL-1β-siRNA vs. LPS + KCC2-siRNA | LPS + IL-1β-siRNA vs. LPS + KCC2-siRNA | LPS + IL-1β-siRNA vs. LPS + KCC2-siRNA | 0.2914 |
| B-P7 | P value | 0.0109 |  |  |
|  | Treatment (between columns) | F (3, 26) = 4.542 |  |  |
|  | Tukey's multiple comparisons test | Mean Diff. | 95.00% CI of diff. | Adjusted P Value |
|  | NS + Control-siRNA vs. LPS + Control-siRNA | -0.4322 | -0.8202 to -0.04406 | 0.0249 |
|  | NS + Control-siRNA vs. LPS + IL-1β-siRNA | -0.04885 | -0.4369 to 0.3392 | 0.9855 |
|  | NS + Control-siRNA vs. LPS + KCC2-siRNA | -0.0008245 | -0.4016 to 0.4000 | >0.9999 |
|  | LPS + Control-siRNA vs. LPS + IL-1β-siRNA | 0.3833 | 0.008369 to 0.7582 | 0.0437 |
|  | LPS + Control-siRNA vs. LPS + KCC2-siRNA | 0.4313 | 0.04324 to 0.8194 | 0.0252 |
|  | LPS + IL-1β-siRNA vs. LPS + KCC2-siRNA | 0.04803 | -0.3401 to 0.4361 | 0.9862 |
| B-P14 | P value | 0.0017 |  |  |
|  | Treatment (between columns) | F (3, 26) = 6.694 |  |  |
|  | Tukey's multiple comparisons test | Mean Diff. | 95.00% CI of diff. | Adjusted P Value |
|  | NS + Control-siRNA vs. LPS + Control-siRNA | -0.5364 | -1.008 to -0.06449 | 0.0215 |
|  | NS + Control-siRNA vs. LPS + IL-1β-siRNA | -0.05371 | -0.5634 to 0.4560 | 0.9914 |
|  | NS + Control-siRNA vs. LPS + KCC2-siRNA | 0.2084 | -0.2636 to 0.6803 | 0.6255 |
|  | LPS + Control-siRNA vs. LPS + IL-1β-siRNA | 0.4827 | -0.02703 to 0.9924 | 0.0682 |
|  | LPS + Control-siRNA vs. LPS + KCC2-siRNA | 0.7448 | 0.2729 to 1.217 | 0.0011 |
|  | LPS + IL-1β-siRNA vs. LPS + KCC2-siRNA | 0.2621 | -0.2477 to 0.7718 | 0.5044 |
| B-P30 | P value | 0.6536 |  |  |
|  | Treatment (between columns) | F (3, 20) = 0.5505 |  |  |
|  | Tukey's multiple comparisons test | Mean Diff. | 95.00% CI of diff. | Adjusted P Value |
|  | NS + Control-siRNA vs. LPS + Control-siRNA | 0.1435 | -0.2640 to 0.5510 | 0.7592 |
|  | NS + Control-siRNA vs. LPS + IL-1β-siRNA | 0.1727 | -0.2348 to 0.5802 | 0.6422 |
|  | NS + Control-siRNA vs. LPS + KCC2-siRNA | 0.1289 | -0.2786 to 0.5364 | 0.8125 |
|  | LPS + Control-siRNA vs. LPS + IL-1β-siRNA | 0.02922 | -0.3783 to 0.4368 | 0.9970 |
|  | LPS + Control-siRNA vs. LPS + KCC2-siRNA | -0.01461 | -0.4221 to 0.3929 | 0.9996 |
|  | LPS + IL-1β-siRNA vs. LPS + KCC2-siRNA | -0.04383 | -0.4514 to 0.3637 | 0.9902 |

Supplementary table 10: Statistical information for Supplementary figure 6.

| P7 | P value | <0.0001 |  |  |
| --- | --- | --- | --- | --- |
|  | Treatment (between columns) | F (3, 20) = 16.47 |  |  |
|  | Tukey's multiple comparisons test | Mean Diff. | 95.00% CI of diff. | Adjusted P Value |
|  | NS + Control-siRNA vs. LPS + Control-siRNA | -1.044 | -1.526 to -0.5610 | <0.0001 |
|  | NS + Control-siRNA vs. LPS + IL-1β-siRNA | -0.07178 | -0.5543 to 0.4108 | 0.9750 |
|  | NS + Control-siRNA vs. LPS + KCC2-siRNA | -0.6419 | -1.124 to -0.1594 | 0.0068 |
|  | LPS + Control-siRNA vs. LPS + IL-1β-siRNA | 0.9717 | 0.4892 to 1.454 | <0.0001 |
|  | LPS + Control-siRNA vs. LPS + KCC2-siRNA | 0.4016 | -0.08093 to 0.8841 | 0.1247 |
|  | LPS + IL-1β-siRNA vs. LPS + KCC2-siRNA | -0.5701 | -1.053 to -0.08759 | 0.0170 |
| P14 | P value | 0.0016 |  |  |
|  | Treatment (between columns) | F (3, 20) = 7.408 |  |  |
|  | Tukey's multiple comparisons test | Mean Diff. | 95.00% CI of diff. | Adjusted P Value |
|  | NS + Control-siRNA vs. LPS + Control-siRNA | -0.4430 | -0.7426 to -0.1435 | 0.0026 |
|  | NS + Control-siRNA vs. LPS + IL-1β-siRNA | -0.05339 | -0.3530 to 0.2462 | 0.9584 |
|  | NS + Control-siRNA vs. LPS + KCC2-siRNA | -0.2826 | -0.5822 to 0.01691 | 0.0687 |
|  | LPS + Control-siRNA vs. LPS + IL-1β-siRNA | 0.3897 | 0.09009 to 0.6892 | 0.0081 |
|  | LPS + Control-siRNA vs. LPS + KCC2-siRNA | 0.1604 | -0.1392 to 0.4600 | 0.4568 |
|  | LPS + IL-1β-siRNA vs. LPS+KCC2-siRNA | -0.2293 | -0.5288 to 0.07030 | 0.1741 |
| P30 | P value | 0.0013 |  |  |
|  | Treatment (between columns) | F (3, 20) = 7.678 |  |  |
|  | Tukey's multiple comparisons test | Mean Diff. | 95.00% CI of diff. | Adjusted P Value |
|  | NS + Control-siRNA vs. LPS + Control-siRNA | -0.4916 | -0.7889 to -0.1943 | 0.0009 |
|  | NS + Control-siRNA vs. LPS+IL-1β-siRNA | -0.1289 | -0.4262 to 0.1684 | 0.6257 |
|  | NS + Control-siRNA vs. LPS + KCC2-siRNA | -0.2063 | -0.5036 to 0.09100 | 0.2428 |
|  | LPS + Control-siRNA vs. LPS + IL-1β-siRNA | 0.3627 | 0.06540 to 0.6600 | 0.0134 |
|  | LPS + Control-siRNA vs. LPS + KCC2-siRNA | 0.2853 | -0.01200 to 0.5826 | 0.0628 |
|  | LPS + IL-1β-siRNA vs. LPS + KCC2-siRNA | -0.07740 | -0.3747 to 0.2199 | 0.8844 |

Supplementary table 11: Statistical information for Supplementary figure 7.

| P7 | P value | 0.0161 |  |  |
| --- | --- | --- | --- | --- |
|  | Treatment (between columns) | F (3, 20) = 4.365 |  |  |
|  | Tukey's multiple comparisons test | Mean Diff. | 95.00% CI of diff. | Adjusted P Value |
|  | NS + Control-siRNA vs. LPS + Control-siRNA | -0.1969 | -0.3761 to -0.01777 | 0.0280 |
|  | NS + Control-siRNA vs. LPS + IL-1β-siRNA | -0.04484 | -0.2240 to 0.1343 | 0.8957 |
|  | NS + Control-siRNA vs. LPS + KCC2-siRNA | 0.005192 | -0.1740 to 0.1844 | 0.9998 |
|  | LPS + Control-siRNA vs. LPS + IL-1β-siRNA | 0.1521 | -0.02707 to 0.3313 | 0.1144 |
|  | LPS + Control-siRNA vs. LPS + KCC2-siRNA | 0.2021 | 0.02296 to 0.3813 | 0.0235 |
|  | LPS + IL-1β-siRNA vs. LPS + KCC2-siRNA | 0.05003 | -0.1291 to 0.2292 | 0.8619 |
| P14 | P value | 0.0061 |  |  |
|  | Treatment (between columns) | F (3, 20) = 5.556 |  |  |
|  | Tukey's multiple comparisons test | Mean Diff. | 95.00% CI of diff. | Adjusted P Value |
|  | NS + Control-siRNA vs. LPS + Control-siRNA | -0.3980 | -0.7132 to -0.08281 | 0.0103 |
|  | NS + Control-siRNA vs. LPS + IL-1β-siRNA | -0.06564 | -0.3808 to 0.2495 | 0.9361 |
|  | NS + Control-siRNA vs. LPS + KCC2-siRNA | -0.01490 | -0.3301 to 0.3003 | 0.9991 |
|  | LPS + Control-siRNA vs. LPS + IL-1β-siRNA | 0.3324 | 0.01717 to 0.6475 | 0.0365 |
|  | LPS + Control-siRNA vs. LPS + KCC2-siRNA | 0.3831 | 0.06791 to 0.6983 | 0.0138 |
|  | LPS + IL-1β-siRNA vs. LPS + KCC2-siRNA | 0.05074 | -0.2644 to 0.3659 | 0.9687 |
| P30 | P value | 0.9788 |  |  |
|  | Treatment (between columns) | F (3, 20) = 0.06291 |  |  |
|  | Tukey's multiple comparisons test | Mean Diff. | 95.00% CI of diff. | Adjusted P Value |
|  | NS + Control-siRNA vs. LPS + Control-siRNA | -0.02358 | -0.3593 to 0.3122 | 0.9972 |
|  | NS + Control-siRNA vs. LPS + IL-1β-siRNA | 0.02679 | -0.3090 to 0.3625 | 0.9959 |
|  | NS + Control-siRNA vs. LPS + KCC2-siRNA | 0.01186 | -0.3239 to 0.3476 | 0.9996 |
|  | LPS + Control-siRNA vs. LPS + IL-1β-siRNA | 0.05037 | -0.2854 to 0.3861 | 0.9744 |
|  | LPS + Control-siRNA vs. LPS + KCC2-siRNA | 0.03545 | -0.3003 to 0.3712 | 0.9907 |
|  | LPS + IL-1β-siRNA vs. LPS + KCC2-siRNA | -0.01493 | -0.3507 to 0.3208 | 0.9993 |
