# Supplementary material for: Severe inflammation in new-borns induces long-term cognitive impairment by activation of IL-1β/KCC2 signaling during early development
Source: BMC Med. 2022 Jul 27;20:235. doi: 10.1186/s12916-022-02434-w (PMC9327322; doi:10.1186/s12916-022-02434-w)
Supplement: Supplementary file 3 — Additional file 3: Fig. S1-S7. Fig. S1: MWM task and FC test for male and female rats after neonatal inflammation. Fig. S2: Western blotting results showing the protein levels of hippocampal IL-1β at P7, P14, and P30 after LPS exposure. Fig. S3: Representative images showing the fluorescence carried by IL-1β-siRNA or KCC2-siRNA. Fig. S4: The mRNA and protein levels of hippocampal KCC2 with development in rats of both sexes. Fig. S5: The mRNA levels of hippocampal IL-1β and KCC2 in P7, P14, and P30 rats after siRNA injection. Fig. S6: The protein levels of hippocampal IL-1β in P7, P14, and P30 rats after siRNA injection. Fig. S7: The protein levels of hippocampal KCC2 in P7, P14, and P30 rats after siRNA injection. [file 12916_2022_2434_MOESM3_ESM.docx]

**Supplementary figures and legends**


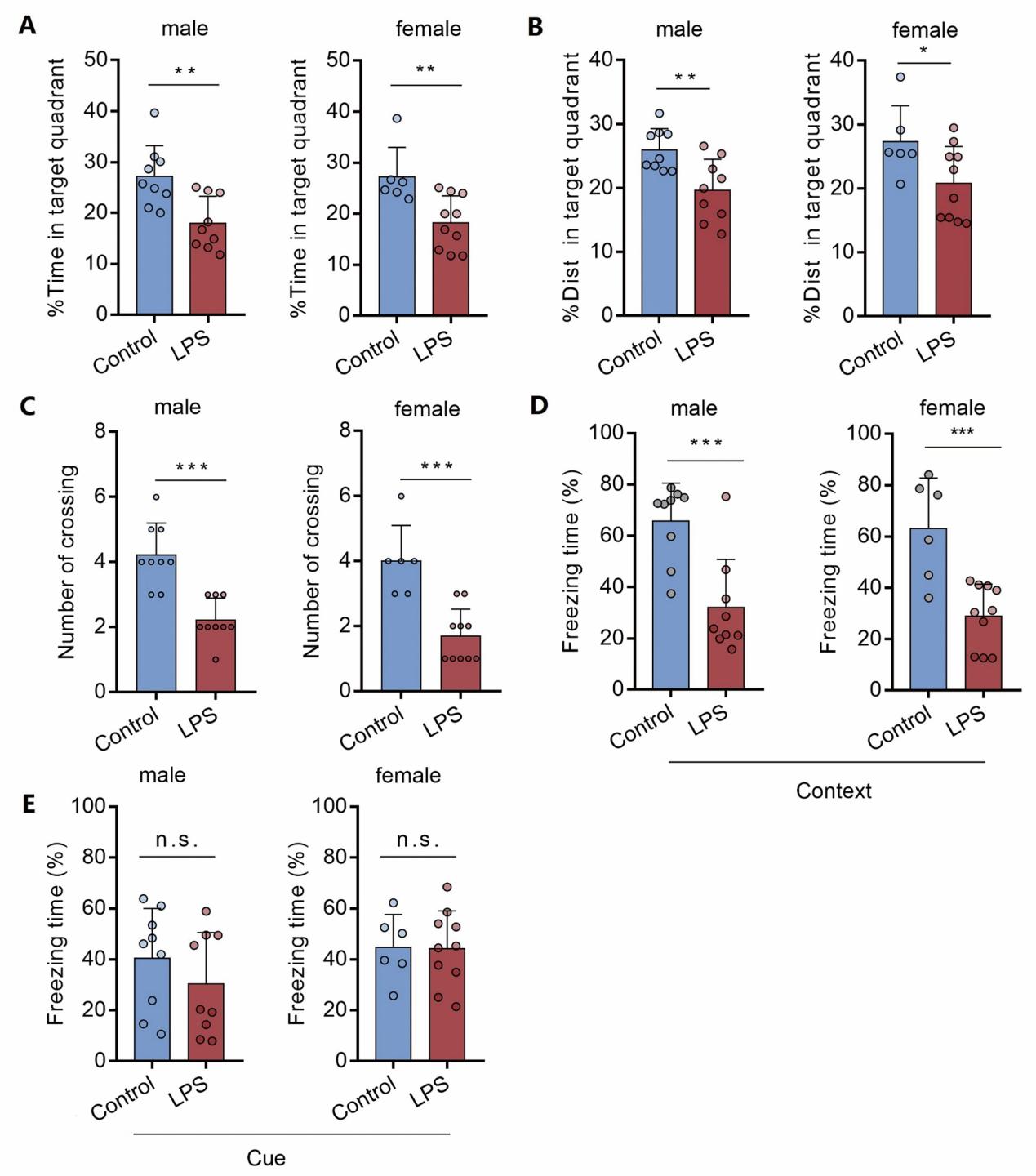


**Supplementary figure 1.** (**A**) The time spent in the target quadrant for male (n = 9) and female rats (n = 6-10). (**B**) Distance spent in the target quadrant for male (n = 9) and female rats (n = 6-10). (**C**) Number of platform crossings for male (n = 9) and female rats (n = 6-10). (**D**) The freezing time of rats in the context FC test for male (n = 9) and female rats (n = 6-10). (**E**) The freezing time of rats in the cued FC test for male (n = 9) and female rats (n = 6-10). LPS: lipopolysaccharide; FC: fear conditioning; Panels A, B, C, D, and E were compared by unpaired two-tailed Student’s t test; * P < 0.05, ** P < 0.01, and *** P < 0.001, n.s.: no significance; Error bars indicate SD.

**
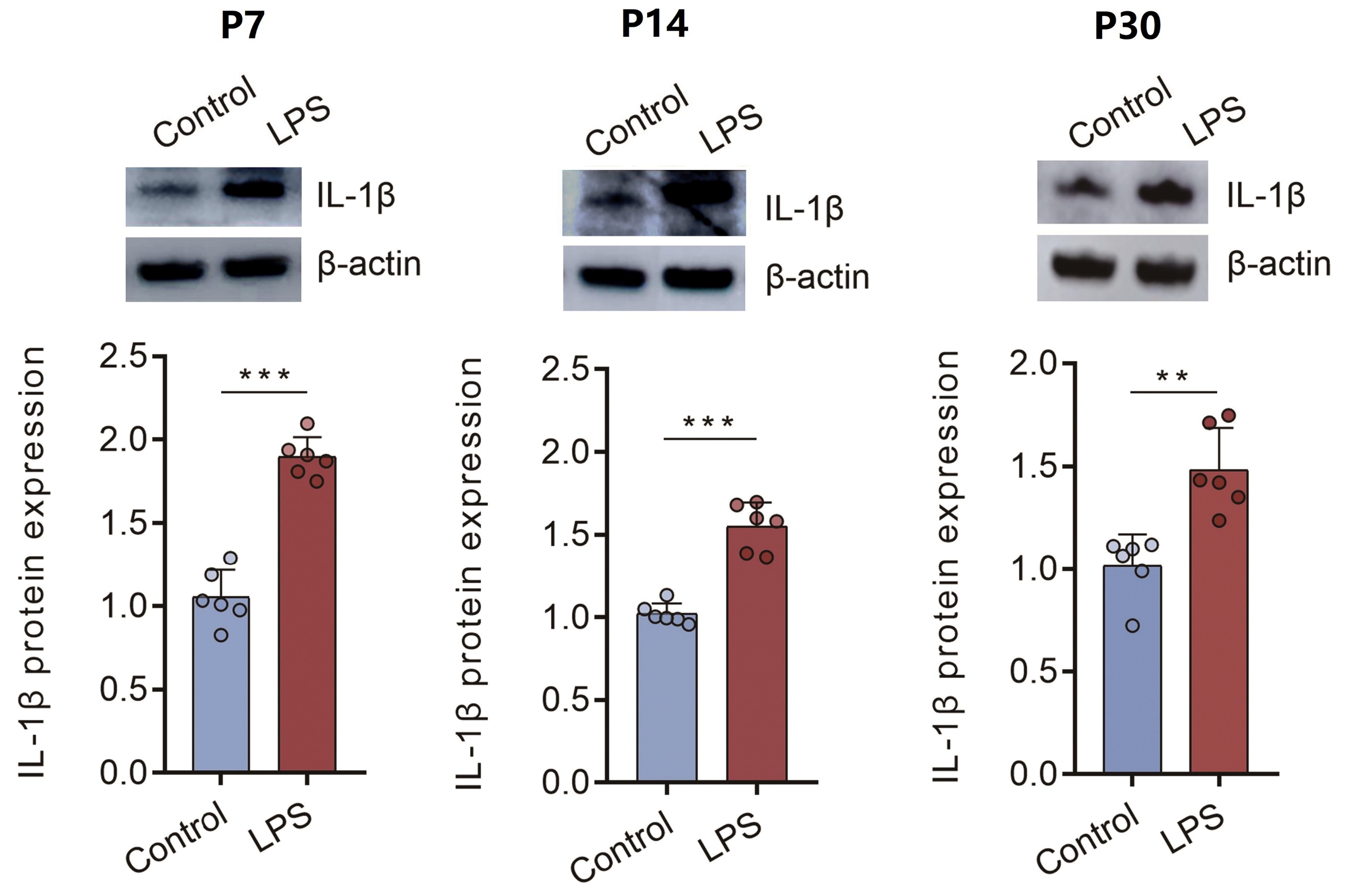
**

**Supplementary figure 2.** Western blot results showing the protein levels of hippocampal IL-1β at P7 (left panel, n = 6), P14 (middle panel, n = 6), and P30 (right panel, n = 6) after LPS exposure. LPS: lipopolysaccharide; Data were compared by unpaired two-tailed Student’s t test; ** P < 0.01, ^***^ *P* < 0.001; Error bars indicate SD.

**
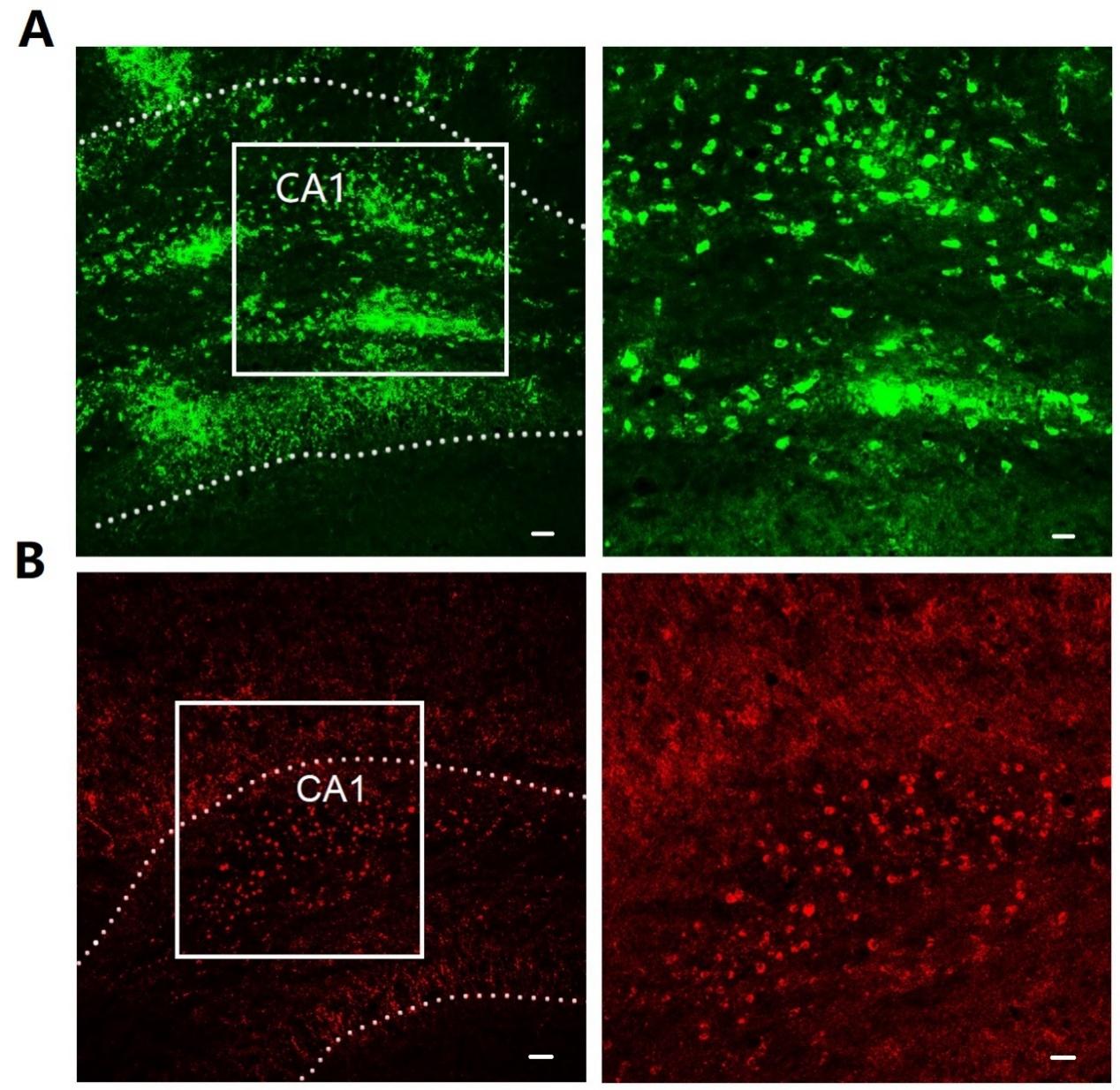
**

**Supplementary figure 3. (A-B)** Representative images showing the ﬂuorescence carried by IL-1β-siRNA (A) or KCC2-siRNA (B). The right panel (scale bar: 25 μm) were enlarged from the white square of the left panel (scale bar: 50 μm).


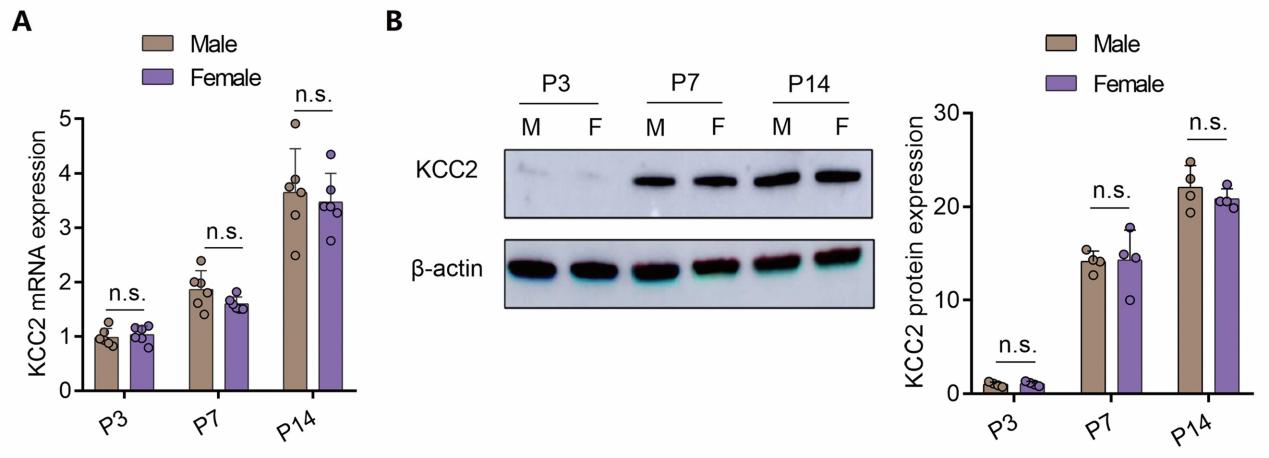


**Supplementary figure 4. (A)** The mRNA levels of hippocampal KCC2 (n = 6) with development in rats of both sexes. **(B)** The protein levels of hippocampal KCC2 (n = 4) with development in rats of both sexes. M: male, F: female; Panels A-B were compared by unpaired two-tailed Student’s t test; n.s.: no significance; Error bars indicate SD.


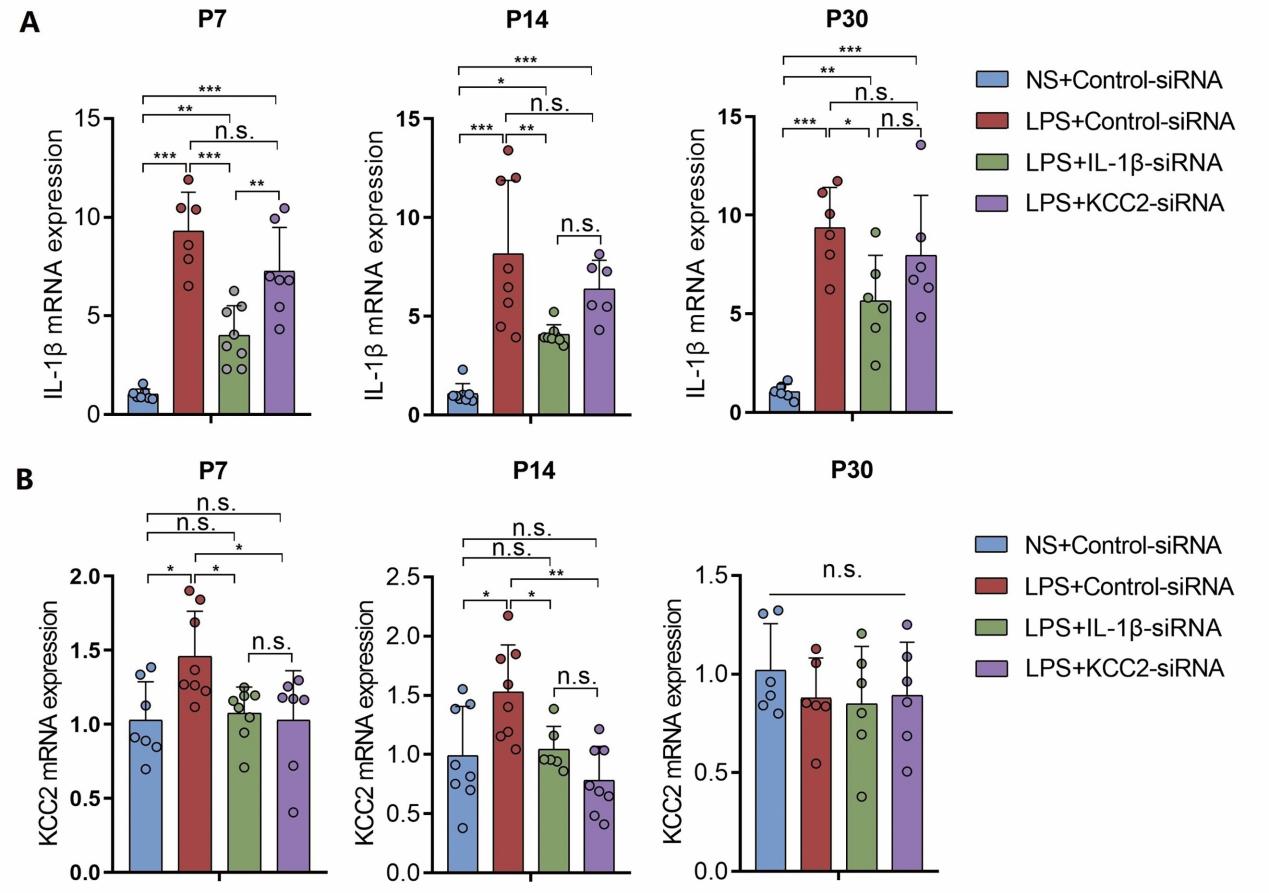


**Supplementary figure 5. (A)** The mRNA levels of hippocampal IL-1β in P7 (left panel, n = 6-8), P14 (middle panel, n = 6-8), and P30 (right panel, n = 6) rats after siRNA injection. **(B)** The mRNA levels of hippocampal KCC2 in P7 (left panel, n = 7-8), P14 (middle panel, n = 6-8), and P30 (right panel, n = 6-8) rats after siRNA injection. LPS: lipopolysaccharide; NS: normal saline; Panels A and B were compared by one-way ANOVA with repeated measures followed by a Tukey *post hoc* test; ^*^ *P* < 0.05, ^**^ *P* < 0.01, and ^***^ *P* < 0.001, n.s.: no significance; Error bars indicate SD.


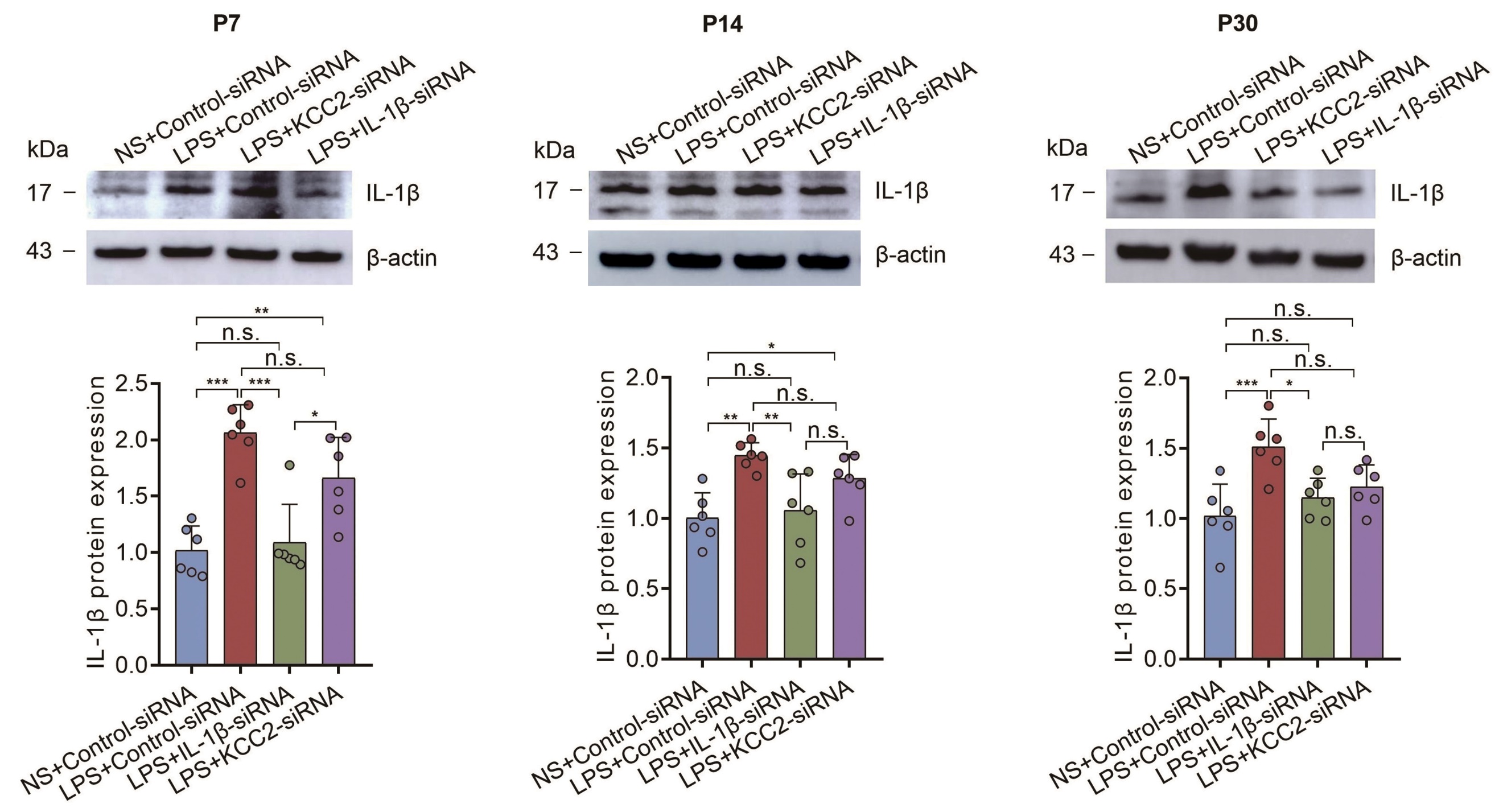


**Supplementary figure 6.** The protein levels of hippocampal IL-1β in P7 (left panel, n = 6), P14 (middle panel, n = 6), and P30 (right panel, n = 6) rats after siRNA injection. LPS: lipopolysaccharide; NS: normal saline; Data were compared by one-way ANOVA with repeated measures followed by a Tukey *post hoc* test; ^*^ *P* < 0.05, ^**^ *P* < 0.01, and ^***^ *P* < 0.001, n.s.: no significance; Error bars indicate SD.


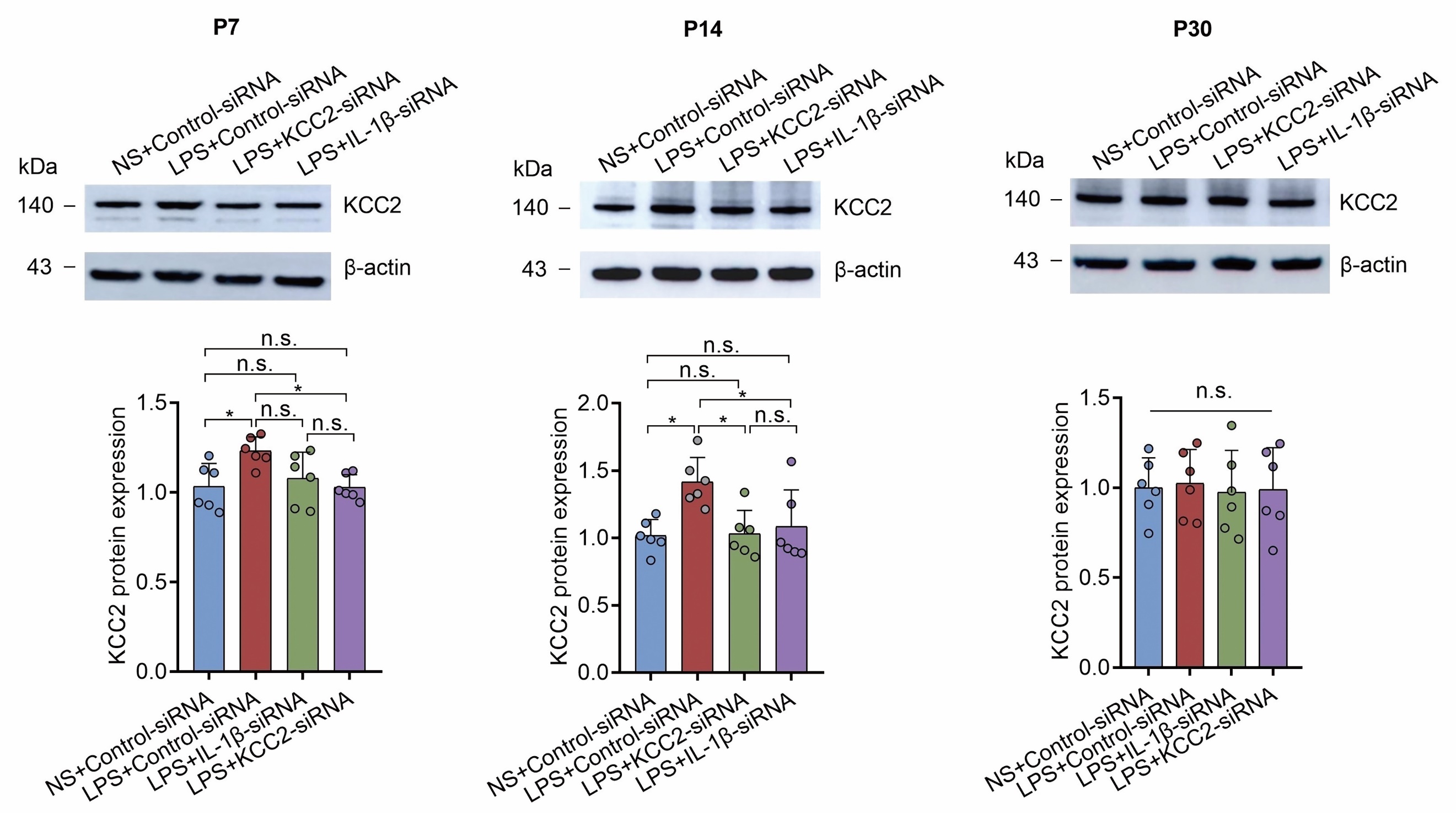


**Supplementary figure 7.** The protein levels of hippocampal KCC2 in P7 (left panel, n = 6), P14 (middle panel, n = 6), and P30 (right panel, n = 6) rats after siRNA injection. LPS: lipopolysaccharide; NS: normal saline; Data were compared by one-way ANOVA with repeated measures followed by a Tukey *post hoc* test; ^*^ *P* < 0.05, n.s.: no significance; Error bars indicate SD.
